# Taxonomic Status, Phylogenetic Affinities and Genetic Diversity of a Presumed Extinct Genus, *Paraisometrum* W.T. Wang (Gesneriaceae) from the Karst Regions of Southwest China

**DOI:** 10.1371/journal.pone.0107967

**Published:** 2014-09-24

**Authors:** Wen-Hong Chen, Yu-Min Shui, Jun-Bo Yang, Hong Wang, Kanae Nishii, Fang Wen, Zhi-Rong Zhang, Michael Möller

**Affiliations:** 1 Key Laboratory for Plant Diversity and Biogeography of East Asia, Kunming Institute of Botany, Chinese Academy of Sciences, Kunming, Yunnan, China; 2 Plant Germplasm and Genomics Center, Germplasm Bank of Wild Species, Kunming Institute of Botany, Chinese Academy of Sciences, Kunming, Yunnan, China; 3 Science Division, Royal Botanic Garden Edinburgh, Edinburgh, Scotland, United Kingdom; 4 Guangxi Institute of Botany, Guangxi Zhuang Autonomous Region and Chinese Academy of Sciences, Guilin, Guangxi, China; 5 University of the Chinese Academy of Sciences, Beijing, China; St. Petersburg Pasteur Institute, Russian Federation

## Abstract

**Background:**

The karst regions in South China have an abundance of endemic plants that face high extinction risks. The Chinese Gesneriaceae endemic *Paraisometrum mileense* ( = *Oreocharis mileensis*), was presumed extinct for 100 years. After its re-discovery, the species has become one of five key plants selected by the Chinese forestry government to establish a new conservation category for plants with extremely small populations. For conservation purposes, we studied the phylogenetic and population genetic status of *P. mileense* at the three only known localities in Guangxi, Guizhou and Yunnan.

**Methodology/Principal Findings:**

We collected 64 samples (52 species) of *Oreocharis* and 8 samples from three provinces of *P. mileense* and generated molecular phylogenies, and inferred that *P. mileense* represents a relatively isolated and derived taxonomic unit within *Oreocharis*. Phylogeographic results of 104 samples of 12 populations of *P. mileense* indicated that the populations in Yunnan have derived from those in Guangxi and Guizhou. Based on AFLP data, the populations were found to harbor low levels of genetic diversity (*He* = 0.118), with no apparent gradient across the species’ range, a restricted gene flow and significant isolation-by-distance with limited genetic differentiation among the populations across the three provinces (*F*_ST_ = 0.207, *P*<0.001). The 10 populations in Yunnan were found to represent two distinct lineages residing at different altitudes and distances from villages.

**Conclusion/Significance:**

The low levels of genetic diversity found in *P. mileense* are perhaps a consequence of severe bottlenecks in the recent past. The distribution of the genetic diversity suggests that all populations are significant for conservation. Current *in situ* and *ex situ* measures are discussed. Further conservation actions are apparently needed to fully safeguard this conservation flagship species. Our work provides a model of an integrated study for the numerous endemic species in the karst regions with extremely small populations.

## Introduction

**Figure 1:**
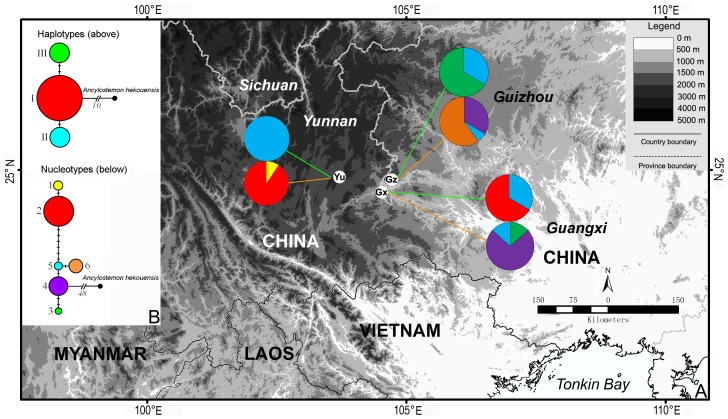
Map of localities of *Paraisometrum mileense* in Yunnan, Guangxi and Guizhou. A. Pie charts for haplotypes (above) and nucleotypes (below) are given for the three main geographical distribution areas. B. Median-joining networks of chloroplast haplotypes based on *trn*LF and *mat*K sequences (above) and of ITS nucleotypes (below) for *P. mileense* samples rooted on its closest relative *Ancylostemon hekouensis*.

**Figure 2:**
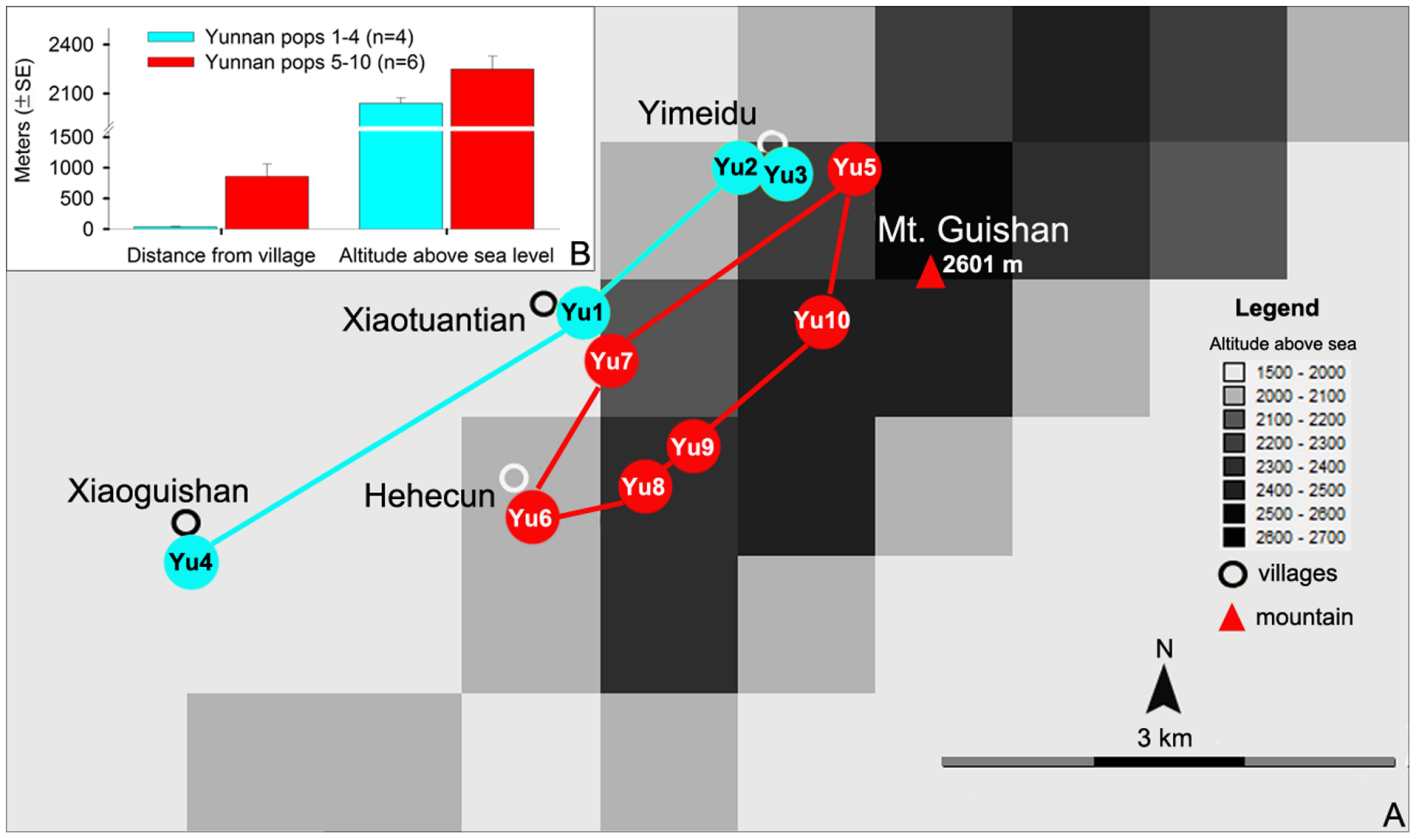
Populations of *Paraisometrum mileense* in Yunnan. A. Distribution of the ten populations analysed. B. Graphs of distance from village (left) and the average altitude (right) of populations 1–4 (light blue) and 5–10 (red).

*Paraisometrum mileense* W.T.Wang was until relatively recently regarded as extinct in the wild because it had not been recollected for more than 100 years since its type specimen collection in 1906 [1], [2]. In 2006, the plant was rediscovered in Shilin county, next to Mile county, Yunnan, where the type specimen was collected [1], [3]. It was described as critically endangered (CR), possessing only 101–1000 individuals in a single population [4]. Interestingly, already in 2009, a second locality had been discovered in Longlin county in Guangxi, the province neighbouring Yunnan to the East [5], and soon afterwards, a third location, in Xingyi, in Guizhou province to the North of Guangxi, had been found [6] (Fig. 1), tripling the number of occurrence points. The total number of plants was estimated at >30,000 individuals [7], but up to 2010, detailed fieldwork by some of the authors (YMS and WHC) estimated the number to be significantly lower, with 630 mature plants in Yunnan, 150 in Guangxi, and 60 in Guizhou (Table 1). Incidentally, approximately 70–80% of the seedlings and young individuals died, and 50–60% of mature individuals sustained damage due to an extreme drought that occurred from 2010 to 2011 in the karst region in Yunnan where *P. mileense* grows (Fig. 2). This might partially explain the discrepancy in reported plant numbers, and illustrates the great vulnerability of the species to even short term climate fluctuations. Until recently, only the occurrence point in Guangxi was located in a provincial nature reserve (established in 2005), while the other two were outside protected areas. In 2011, however, the Chinese government set up a small reserve in Shilin, Yunnan, to protect the species at this primary rediscovery point (Fig. 2). The species is also one of five selected key plants to be used to establish a new category of protected areas in China for plant species with extremely small populations (PSESP) [4].

**Table 1:** Detailed locality information for the 12 populations of *Paraisometrum mileense* used for AFLP analysis.

Population code	Locality name	Forest type	Position on slope	Altitude (m)	Distance (m) from village	Population size*	Area size (ha)	Sample number	Individual sample no
Yu1	Yunnan, Shilin, Guishan	secondary	lower	2001	25	46	0.40	5	Yu1a–e
Yu2	Yunnan, Shilin, Guishan	secondary	lower	2083	40	50	0.20	5	Yu2a–e
Yu3	Yunnan, Shilin, Guishan	secondary	lower	2112	20	52	0.25	5	Yu3a–e
Yu4	Yunnan, Shilin, Guishan	secondary	lower	1970	60	100	0.65	15	Yu4a–n
Yu5	Yunnan, Shilin, Guishan	primary	lower-middle	2149	800	76	0.25	8	Yu5a–e
Yu6	Yunnan, Shilin, Guishan	primary	lower-middle	2018	40	84	0.26	5	Yu6a–f
Yu7	Yunnan, Shilin, Guishan	primary	lower-middle	2077	600	120	1.20	5	Yu7a–e
Yu8	Yunnan, Shilin, Guishan	primary	middle-upper	2349	1000	32	0.20	5	Yu8a–e
Yu9	Yunnan, Shilin, Guishan	primary	upper	2412	1200	36	0.15	5	Yu9a–e
Yu10	Yunnan, Shilin, Guishan	primary	upper	2496	1500	34	0.16	10	Yu10a–i
Gx11	Guangxi, Longlin, Yacha	primary	upper	1183	1500	150	0.80	19	Gx11a–s
Gz12	Guizhou, Xingyi, Jingnan	primary	middle-upper	1405	1000	60	0.60	17	Gz12a–q

* - approximate number of mature plants per population based on data from August 2010.

Until 2011, *Paraisometrum* W.T.Wang was regarded as a monotypic genus, but was then included in an enlarged genus *Oreocharis* Benth. as *O. mileensis* (W.T.Wang) Mich.Möller & A.Weber [8]. Irrespective of this recent inevitable taxonomic change, the species represents a highly threatened taxon. Furthermore, under its old name, it has received considerable attention as a strong flagship species for plant conservation in China, and we therefore use its original name here in this context, for consistency with current conservation initiatives, such as the establishment of the above new conservation category in China [4].

To be able to devise meaningful conservation strategies, knowledge of the taxonomic status, closest congeners and the level and distribution of genetic diversity within a taxon is essential [9]–[11]. Even though *P. mileense* has been included in phylogenetic analyses previously, its exact phylogenetic affinities in *Oreocharis* are still unclear. While it was clearly shown in the phylogenetic analyses that the species has evolved from within the enlarged *Oreocharis*, it fell on a polytomy with species of the *hitherto* genera *Ancylostemon* Craib, *Briggsia* Craib, *Isometrum* Craib, *Opithandra* B.L.Burtt and *Tremacron* Craib [8], [12], [13]. The generic characters to establish *Paraisometrum* are the presence of four upper corolla lobes, and one lower lobe of the pentamerous flowers [2]. Since this characteristic also occurs in other species of the newly defined *Oreocharis* (e.g. *O. saxatilis* (Hemsl.) Mich.Möller & A.Weber = *Ancylostemon saxatilis* Hemsl.) [14]–[17], the present work will address the species delineation of *P. mileense*. Furthermore, the plants in Guangxi and Guizhou appear to possess some floral features different from those in Yunnan (pers. obs. YMS and WHC). However, only two samples of *P. mileense*, one from Yunnan and one from Guangxi, were included in the most comprehensive study of *Oreocharis* to date [12]. This is not enough to test the taxon coherence in the light of modern approaches such as DNA barcoding [18]–[20]. Clearly, more molecular work was needed to include samples from all three occurrence points and from diverse species within the enlarged *Oreocharis*.

There are many Gesneriaceae species endemic to the karst region in China, typically with small populations [7], such as *Primulina tabacum* Hance. This species occurs in only four populations distributed in Guangdong and Hunan with less than 1,000 plants in each [21]. Despite the relatively low plant number, a surprisingly high genetic diversity was found within the populations. Additionally, because of the long inter-population distances, a disruption of gene flow resulted in high population differentiation [10]. From a conservation perspective, the situation in *P. tabacum* was seen as ‘a window of opportunity’ to preserve a high level of extant genetic variation in the species. To determine whether a similar situation is present in *P. mileense*, we generated population genetic data using amplified fragment length polymorphisms (AFLPs) for individuals from the three localities in Guangxi, Guizhou and Yunnan. AFLP is a powerful tool for generating data from multiple loci for the detection of genetic variation without the need for pre-existing knowledge of genomic sequences [22], [23], and have been successfully applied to small and relict populations (e.g. [10], [24]–[27]), and a range of suitable analytical packages are available for these dominant markers (e.g. [27]).

Thus, our main aims were fourfold, to phylogenetically analyse sequence data, from the chloroplast intron–spacer sequences of *trn*LF, and the nuclear ribosomal internal transcribed spacer regions (nrITS), to a) test the taxonomic status of the species, b) to determine the phylogenetic position and relationships of *P. mileense* within the enlarged *Oreocharis*, c) to reconstruct the phylogeographic history of the *P. mileense* populations using *trn*LF, *mat*K, and ITS sequence data for individuals from 12 populations (or sub-populations) from the only three localities in Guangxi, Guizhou and Yunnan, and to acquire and analyse AFLP data for population samples to d) determine the levels of genetic diversity and differentiation of this species to assess its conservation requirements.

## Materials and Methods

### Ethics Statement

Of all sampled species, the target species *Paraisometrum mileense* is listed as one of five selected key plants to be used to establish a new category of protected areas in China for plant species with extremely small populations (PSESP). Approvals and permission for field studies were obtained from the Yunnan Forestry Bureau, China (permit no. [2011]–115). The GPS data are sensitive and are not provided for the protection of the plants according to the Yunnan forestry administrative request in the permit. However, we gave their approximate location in Fig. 1.

### Study Materials

For the phylogenetic study, we expanded the ingroup sampling from 38 *Oreocharis* species plus one variety (41 samples) [12] to 52 species plus 2 varieties (73 samples), among which 11 species covered at least two population samples each. Of *P. mileense*, we included three samples each from Guangxi and Guizhou and two samples from Yunnan (Table S1). Thus, the *Oreocharis* ingroup sampling covered about 2/3 of the genus (53 out of about 80 species) [12]. The outgroup included 14 samples across six genera of straight-fruited advanced Asiatic and Malesian Gesneriaceae of subtribe Didymocarpinae (*sensu* [28]), with two *Didymocarpus* Wall. samples to root the trees [8], [12].

The *Paraisometrum* materials for the phylogeographic analyses included 43 individuals from Yunnan (from 10 populations or sub-populations), 15 from the Guangxi population and 15 from the Guizhou population, and for the AFLP analysis 68 individuals from Yunnan (from 10 populations, 5–15 samples per population), 19 from the Guangxi population and 17 from the Guizhou population (Fig. 1; Table 1).

### Molecular Methods

Genomic DNA was extracted from silica-gel-dried leaves collected in the field following a modified CTAB method [29], [30]. The PCR primers for ITS were ‘ITS5’ and ‘ITS4’ [31], for *trn*LF ‘C’ and ‘F’ [32], and for *mat*K ‘3F’ and ‘1R’ [30]. PCR was performed in reactions containing 30–50 ng genomic DNA, 0.3 µl of each primer (5 µM/µl), 10 µl 2×*Taq* PCR MasterMix (Tiangen Biotech Co., Ltd, Beijing China: 0.1 U *Taq* polymerase/µl, 0.5 nM of each dNTP, 20 mM Tris-HCl (pH 8.3), 100 mM KCl, 3 mM MgCl_2_) and ddH_2_O to make up 20 µl. PCR amplifications were conducted under the following profile: 95°C for 3 min followed by 35 cycles at 94°C for 30 s, at the annealing temperature specific for each primer pair for 30 s (ITS: 55°C; *trn*LF and *mat*K: 52°C), 72°C for 1 min, and a final extension step at 72°C for 5 min. After PCR amplicon purification, Sanger sequencing was carried out in 6 µl reactions containing 0.5 µl PCR product, 0.3 µl primer (5 µM/µl), 1.05 µl SeqBuffer, 0.3 µl BigDye Terminator Mix (Applied Biosystems, Foster City, USA) and 3.85 µl distilled H_2_O. Sequencing reactions were cycled under the following conditions: 32 cycles at 96°C for 10 s, 50°C for 5 s, and 60°C for 3 min, and the products analysed on an ABI 3730×l sequencer (Applied Biosystems, Foster City, USA). The newly acquired sequences have been submitted to GenBank.

AFLP was performed based on Vos et al. [23]. Restriction endonuclease enzyme digestion and link reactions were performed in 20 µl reactions containing 4 µl template DNA (50 ng/µl), 1 µl Adaptor, 2 µl *Pst*I/*Mse*I, 2.5 µl 10× reaction buffer, 2.5 µl ATP (10 mM), 1 µl T4 Ligase, 7 µl distilled H_2_O. The solution was centrifuged for a few minutes after stirring, and incubated at 37°C for 5 h, then at 8°C for 4 h and then at 4°C overnight. Pre-amplification reactions were performed containing 2 µl template DNA, 1 µl Pre-ampmix, 0.5 µl dNTPs (Table 2), 2.5 µl 10× PCR buffer, 0.5 µl *Taq* polymerase, and 18.5 µl ddH_2_O. PCR amplifications were conducted with the profile: 94°C for 2 min, followed by 30 cycles at 94°C for 30 s, 56°C for 30 s, 72°C for 80 s, with a final extension at 72°C for 5 min, and terminated at 4°C. Eight selective amplification primers were used for each *Pst*I and *Mse*I (Table 2). The selective amplification reactions were performed in 25 µl reactions containing 2 µl 1∶20 diluted preamplification product, 2.5 µl 10× PCR buffer, 0.5 µl dNTP, 1 µl *Pst*I primer, 1 µl *Mse*I primer, 0.5 µl *Taq* polymerase, and 17.5 µl ddH_2_O. PCR amplifications were conducted as follows: 94°C for 30 s, 65°C for 30 s, 72°C for 80 s for the first cycle, followed by 12 cycles with progressively decreasing annealing temperature by 0.7°C each cycle starting from 65°C to 56.6°C, which was followed by 23 cycles of 94°C for 30 s, 55°C for 30 s, 72°C for 80 s, with a final extension step at 72°C for 5 min, and then cooled to 4°C. The AFLP primers, *Taq* polymerase and dNTPs for PCR were purchased from Dingguochangsheng Biotechnology Co. Ltd. (Beijing, China). The amplified fragments were separated and detected with an ABI Prism 377 sequencer (Applied Biosystems, Foster City, USA). Due to ambiguous banding patterns or obvious PCR failures, 19 additional samples (GX11a-s) collected exclusively from Guangxi province were included (Table 1). The scoring error rate was about 1.3%, determined by replicating AFLP runs for 16 individuals (ca. 15% of all samples analysed) (*cf*. [33]).

**Table 2:** Name and DNA sequences of primers and adaptors used in the AFLP experiments on *Paraisometrum mileense* samples.

	Primer name	Primer sequence
Adaptors	*Pst*I 1	5′ -CTC GTA GAC TGC GTA CAT GCA
	*Pst*I 2	5′ -TGT ACG CAG TCT AC
	*Mse*I 1	5′ -GAC GAT GAG TCC TGA G
	*Mse*I 2	5′ -TAC TCA GGA CTC AT
Pre-amplification primers	*Pst*I	5′ -GAC TGC GTA CAT GCA G
	*Mse*I	5′ -GAT GAG TCC TGA GTA A C
Selective amplification primers		
*Pst*I primers (5 ng/µl)	*Pst*I-1	5′ -GAC TGC GTA CAT GCA GAA
	*Pst*I-2	5′ -GAC TGC GTA CAT GCA GAC
	*Pst*I-3	5′ -GAC TGC GTA CAT GCA GAG
	*Pst*I-4	5′ -GAC TGC GTA CAT GCA GAT
	*Pst*I-5	5′ -GAC TGC GTA CAT GCA GTA
	*Pst*I-6	5′ -GAC TGC GTA CAT GCA GTC
	*Pst*I-7	5′ -GAC TGC GTA CAT GCA GTG
	*Pst*I-8	5′ -GAC TGC GTA CAT GCA GTT
*Mse*I primers (30 ng/µl)	*Mse*I-1	5′ -GAT GAG TCC TGA GTA ACA A
(marked FAM)	*Mse*I-2	5′ -GAT GAG TCC TGA GTA ACA C
	*Mse*I-3	5′ -GAT GAG TCC TGA GTA ACA G
	*Mse*I-4	5′ -GAT GAG TCC TGA GTA ACA T
	*Mse*I-5	5′ -GAT GAG TCC TGA GTA ACT A
	*Mse*I-6	5′ -GAT GAG TCC TGA GTA ACT C
	*Mse*I-7	5′ -GAT GAG TCC TGA GTA ACT G
	*Mse*I-8	5′ -GAT GAG TCC TGA GTA ACT T

### Phylogenetic Analyses

The phylogenetic analyses were conducted on a matrix containing 86 ITS and *trn*LF sequences. ITS and *trn*LF sequences for 37 samples were newly acquired (Table S1), and for 49 samples retrieved from GenBank. The newly acquired DNA sequences were assembled and trimmed in Sequencher 4.1.4 (Gene Codes, Ann Arbor, MI, USA), and added to, and manually aligned with, the existing matrices. Maximum parsimony (MP) and Bayesian inference (BI) analyses followed Möller et al. [8], [12] and Weber et al. [34], using PAUP* 4.0b10 [35] and MrBayes 3.2.2 [36], [37], respectively.

The partition-homogeneity test in PAUP* (incongruence length difference test [38], [39], on 1,000 replicates of repartitioning with tree bisection-reconnection (TBR) indicated no significant incongruence between the two datasets (*P* = 0.28) and the ITS and *trn*LF data sets were analysed combined. Parsimony was implemented on unordered and unweighted characters and through heuristic tree searches on 10,000 random starting trees with both TBR swapping and MulTrees on. Clade support was obtained as bootstrap indices with 10,000 heuristic replicates of random additions with TBR on and MulTrees off.

The most suitable substitution model for the BI analysis was obtained under the AIC criterion in MrModeltest [40], and was GTR+G for both, *trn*LF and the ITS spacers, and SYM+G for the 5.8S gene. Two independent runs of four MCMC chains of two million generations were run, sampled each 1,000^th^ generation. The first 200 trees (10%) were discarded as burn-in (generations prior to stationarity of likelihood values), and the posterior probabilities (PP) obtained from 50% majority rule consensus trees obtained using the ‘sumt’ command in MrBayes. A low value of the average standard deviation of split frequencies (0.009025), the high correlation of the PP support values and the parallel distribution of variance values found between the two parallel runs of the Bayesian analysis, and the level runs of the split posteriors indicated a good convergence of the MCMC runs (Table S2; Figs. S1–S3).

### Phylogeographic Analyses

Chloroplast haplotypes were determined using combined chloroplast *trn*LF and *mat*K sequence data, and nucleotypes using ITS (GenBank accession numbers *trn*LF: KM062935–KM062942, KM06301–KM063145; *mat*K: KM063008–KM063080; ITS: KM062943–KM063007; KM063175–KM063182). Phylogeographic networks were reconstructed with NETWORK 4.6.1.1 (available at http://www.fluxus-engineering.com). *Ancylostemon hekouensis* Y.M.Shui & W.H.Chen ( = *Oreocharis hekouensis* (Y.M.Shui & W.H.Chen) Mich.Möller & A.Weber) was used to root the networks, based on finding of the phylogenetic analyses here.

### Population Genetic Analyses

The ABI trace files were analysed in GeneScan 3.1 (Applied Biosystems), and only intensive bands between 70 and 500 base pairs in size converted to a binary matrix (1 = band presence, 0 = band absence) (Table S3). Genetic diversity parameters were obtained in GenAlEx 6.5 [41], and included percentage of polymorphic loci (*P*), number of different alleles (*Na*), number of effective alleles (*Ne*), Shannon’s Information index (*SI*), expected heterozygosity (*He*) and unbiased expected heterozygosity (*uHe*). To investigate for patterns among the *Paraisometrum* populations, we used STRUCTURE 2.3.4. [42] to assign individuals to genotypically distinct groups. We used the admixture model and the option for correlated allele frequencies as recommended by Falush et al. [43]. The program was run 14 times for each cluster (*K*) from *K*1 to *K*10. Each run of 100,000 iterations was preceded by 10,000 iterations as burn-in when convergence was achieved. We plotted the mean likelihood for each cluster *L*(*K*) against the cluster number (*K*). To establish the optimal number of clusters, the relationship between *K* and *ΔK*, the second order rate of change of the likelihoods, was plotted [44]. The distribution of genetic variation within and between populations and between regions was analysed in a hierarchical AMOVA in GenAlEx.

To test an isolation-by-distance scenario, a Mantel test was performed on the geographic and *ln* geographic distance *versus* Nei’s genetic distance (*D*) in GenAlEx. For a 3D illustration of spatial relationships of the populations, a principal coordinate analysis (PCoA) was performed using the Jaccard distance in R-pack Le Progiciel R.4.0d10 [45]. To illustrate the genetic relationships between the populations, an unrooted Neighbor Joining (NJ) tree was reconstructed using Nei and Li’s restriction site distances in PAUP*. Branch support was obtained from 1000 NJ bootstrap replicates in PAUP*.

## Results

### Phylogeny

The MP analysis recovered 12 most parsimonious trees of 1538 steps length with a CI of 0.5566 and RI of 0.6982. The genus *Oreocharis* in its new definition formed a strongly supported clade in the strict consensus tree (BS = 100%). *Paraisometrum* was nested deeply within this clade in a derived position with the *P. mileense* samples forming a strongly supported clade (BS = 100%) (Fig. S4). Sister to this clade were two samples of *A. hekouensis*, though with no branch support.

**Figure 3:**
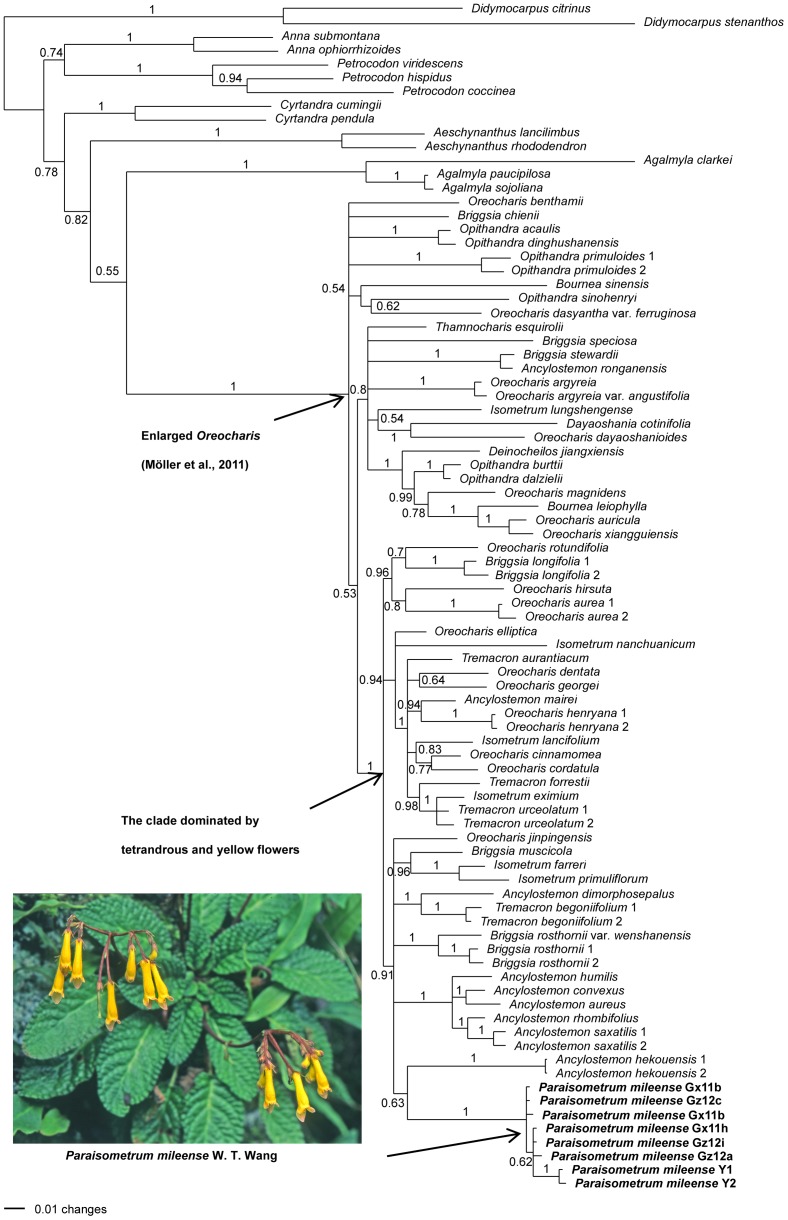
Bayesian inference analysis placing *Paraisometrum mileense* in *Oreocharis*. Tree based on combined ITS and *trn*LF sequence data with average branch lengths and posterior probabilities. Photo of *P. mileense* by Yu-Min Shui.

The BI trees showed the same phylogenetic positions with view to the *Paraisometrum* and *A. hekouensis* samples (Fig. 3), the sister-relationship of the two clades received only low support (PP = 0.63). The BI tree was more resolved both within the enlarged *Oreocharis* and within the *Paraisometrum* clade. *Paraisometrum* was nested deeply in a derived position within the clade dominated by species with yellow tetrandrous flowers (Fig. 3). The populations from Guizhou and Guangxi appeared intermixed in two groups not reflecting their origins (PP = 0.62), while the samples from Yunnan were most similar to each other (PP = 1.0), and somewhat distant to those from the other provinces.

### Phylogeography

Among the 1676 bases in the combined *trn*LF and *mat*K matrix, three positions were variable among the *Paraisometrum* samples, resulting in three chloroplast haplotypes (Fig. 1; Table 3). Haplotype I was the only one in Yunnan and present in the other two provinces, while haplotype II was unique to Guangxi, and haplotype III was a private haplotype in the population from Guizhou (Fig. 1A). The median-joining network, rooted on *A. hekouensis*, placed haplotype I in a central position with haplotypes II and III as peripherals (Fig. 1B).

**Table 3:** Haplotypes and nucleotypes found among the samples of *Paraisometrum mileense.*

Regions	N	Haplotypes			Nucleotypes						
		I	II	III	1	2	3	4	5	6	
Yunnan (Pops1–10)	43	43	–	**–**	4	39	**–**	**–**	**–**	**–**	
[Yunnan (Pops1–4)]	22	22	**–**	**–**	2	20	**–**	**–**	**–**	**–**	
[Yunnan (Pops5–10)]	21	21	**–**	**–**	2	19	**–**	**–**	**–**	**–**	
Guangxi (Pop11)	15	5	10	**–**	**–**	**–**	2	11	2	**–**	
Guizhou (Pop12)	15	5	**–**	10	**–**	**–**	**–**	5	1	9	
Total	73	**–**	**–**	**–**	**–**	**–**	**–**	**–**	**–**	**–**	

Among the 643 bases of the ITS region, eleven positions were variable, giving six different ITS nucleotypes among the samples with types 1 and 2 exclusive to Yunnan, type 4 and 5 shared among the Guangxi and Guizhou populations, while type 3 was private in Guangxi, and type 6 private in Guizhou (Fig. 1A; Table 3). The median-joined network placed type 4 present in Guangxi and Guizhou in the centre, from which first types only present in these two provinces have evolved; the ITS types 1 and 2 present in Yunnan were in a derived position (Fig. 1B).

### Genetic Diversity

At the population level, the AFLP data suggested a low level of genetic diversity of the populations, particularly in Yunnan (Table 4). This is likely a result of the low number of individuals included per populations. At the region level, the data suggested that the Yunnan populations harboured an overall higher level of diversity. For example, the percentage of polymorphic loci was 90.88% in Yunnan, compared to 61.18% in Guangxi and 66.99% in Guizhou. Other genetic diversity indices were also slightly higher, such as the Shannon’s Information index (Yunnan: 0.247, Guangxi: 0.219, Guizhou: 0.235), though the number of effective alleles and heterozygosity indices were very similar in the three provinces.

**Table 4:** Genetic diversity indices based on AFLP data among the 12 populations of *Paraisometrum mileense* analysed.

Population	*N*	*P*	*Na*	*Ne*	*SI*	*He*	*uHe*
Yu1	5	33.37	0.744 (0.023)	1.160 (0.007)	0.155 (0.006)	0.100 (0.004)	0.111 (0.004)
Yu2	5	36.37	0.762 (0.024)	1.172 (0.007)	0.170 (0.006)	0.109 (0.004)	0.121 (0.004)
Yu3	5	33.01	0.712 (0.023)	1.161 (0.007)	0.156 (0.006)	0.100 (0.004)	0.112 (0.004)
Yu4	15	55.85	1.130 (0.024)	1.195 (0.007)	0.200 (0.006)	0.124 (0.004)	0.128 (0.004)
Yu5	8	44.34	0.938 (0.024)	1.202 (0.008)	0.195 (0.006)	0.125 (0.004)	0.133 (0.005)
Yu6	5	28.60	0.660 (0.022)	1.154 (0.007)	0.142 (0.006)	0.093 (0.004)	0.103 (0.004)
Yu7	5	32.95	0.727 (0.023)	1.174 (0.007)	0.162 (0.006)	0.106 (0.004)	0.118 (0.005)
Yu8	5	36.01	0.760 (0.024)	1.194 (0.008)	0.179 (0.006)	0.117 (0.004)	0.130 (0.005)
Yu9	5	43.66	0.891 (0.024)	1.197 (0.007)	0.199 (0.006)	0.126 (0.004)	0.140 (0.004)
Yu10	10	49.17	1.017 (0.024)	1.224 (0.008)	0.214 (0.006)	0.137 (0.004)	0.144 (0.005)
Gx	19	57.99	1.190 (0.024)	1.213 (0.008)	0.212 (0.006)	0.133 (0.004)	0.137 (0.004)
Gz	17	66.99	1.344 (0.023)	1.220 (0.007)	0.235 (0.006)	0.143 (0.004)	0.148 (0.004)
Total	8.7(0.036)	43.19 (3.48)	0.906 (0.007)	1.189 (0.002)	0.185 (0.002)	0.118 (0.001)	0.127 (0.001)
**Mean over Loci for each region**							
Yunnan	68	90.88	1.818 (0.014)	1.231 (0.007)	0.247 (0.006)	0.149 (0.004)	0.150 (0.004)
Guangxi	19	60.75	1.240 (0.024)	1.219 (0.008)	0.219 (0.006)	0.137 (0.004)	0.141 (0.004)
Guizhou	17	67.42	1.352 (0.023)	1.227 (0.007)	0.238 (0.006)	0.146 (0.004)	0.151 (0.004)
**Grand Mean over Loci and pops**							
Total	34.667(0.337)	73.01(9.14)	1.470(0.013)	1.226(0.004)	0.235(0.003)	0.144(0.002)	0.147(0.002)

Values are means (and SE).

N = no. of samples; *P* = percentage of polymorphic loci; *Na* = no. of different alleles; *Ne* = no. of effective alleles; *SI* = Shannon’s Information index; *He* = expected heterozygosity; *uHe* = unbiased expected heterozygosity.

### Genetic Structure

The AMOVA indicated that most genetic diversity resided within the populations of *P. mileense* (79%) and significant genetic differences existed among populations (21%, *F*_ST_ = 0.207, *P*<0.001) (Table 5). When structured for regions, a similar genetic differentiation was observed between the three provinces (12%, *F*_CT_ = 0.120, *P*<0.001) and among populations (12%, *F*_SC_ = 0.144, *P*<0.001) (Table 6).

**Table 5:** Results of an unstructured hierarchical AMOVA on AFLP data of 12 populations of *Paraisometrum mileense.*

Source	df	SS	MS	Est. Var.	Var. (%)	*F* statistics	*P*
Among Pops	11	4914.428	446.766	36.513	21	*F*_ST_ = 0.207	<0.001
Within Pops	92	12907.486	140.299	140.299	79	−	−
Total	103	17821.913	−	176.812	100	−	−

**Table 6:** Results of a structured hierarchical AMOVA on AFLP data of 12 populations of *Paraisometrum mileense*, with three regions, Yunnan (10 pops), Guangxi (1 pop), Guizhou (1 pop).

Source	df	SS	MS	Est. Var.	Var. (%)	*F* statistics	*P*
Among Regions	2	2243.796	1121.898	22.405	12	*F*_CT_ = 0.120	<0.001
Among Pops	9	2670.632	296.737	23.581	13	*F*_SC_ = 0.144	<0.001
Within Pops	92	12907.486	140.299	140.299	75	*F*_ST_ = 0.247	<0.001
Total	103	17821.913	−	186.286	100	−	−

**Figure 4:**
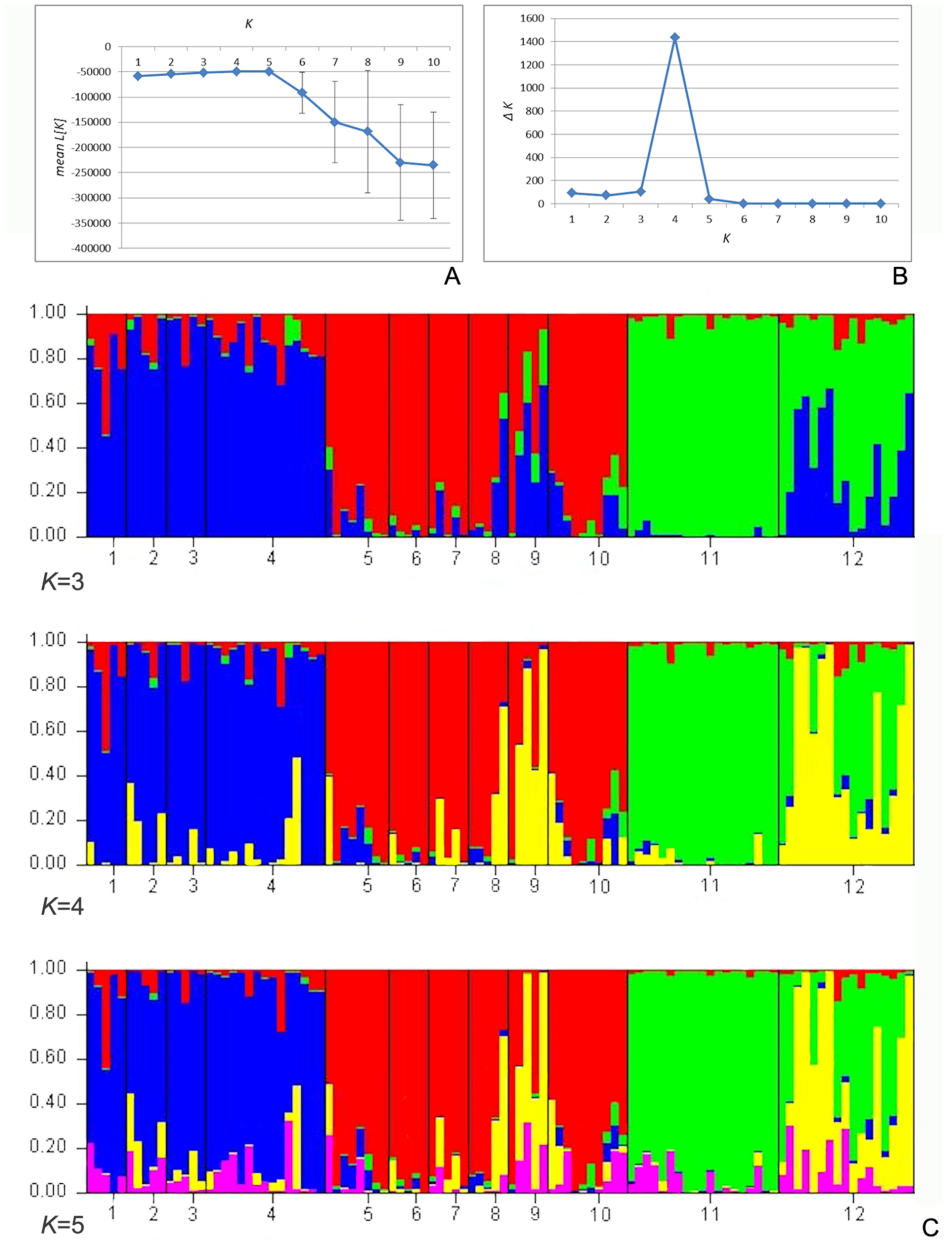
Results of the Bayesian inference STRUCTURE analysis on AFLP data of 12 *Paraisometrum mileense* populations. A. Plot of *K*-clusters *versus* mean (±SD) likelihoods (L[K]). B. *K* plotted against the second order rate of change of the likelihoods (Δ*K*). C. STRUCTURE clustering results for *K* = 3 to 5 as suggested in B. Numbers refer to populations in Table 1. 1–10 = Yunnan, 11 = Guangxi, 12 = Guizhou.

The optimal number of clusters for the STRUCTURE analysis was determined as *K* = 4 (Fig. 4 A and B). The analysis revealed a strong separation between the Yunnan populations 1–4 and 5–10 (Fig. 4C). The Guangxi population (Pop 11, Gx) was very homogeneous and distinct. The Guizhou population (Pop 12, Gz) was a mix of two types, one shared with the Guangxi population and the other individuals similar to a great degree to samples of the Yunnan populations 8 and 9. With *K* = 3, the patterns did not change greatly, except that the latter genotypes were clustering with the populations 1–4 from Yunnan. With *K* = 5, no further change in clustering was observed, the fifth genetic cluster scattering in small proportions among all populations. The Mantel test was significant using the geographic distance (r = 0.398; *P* = 0.01) and *ln* geographic distance (r = 0.383; *P* = 0.03).

**Figure 5:**
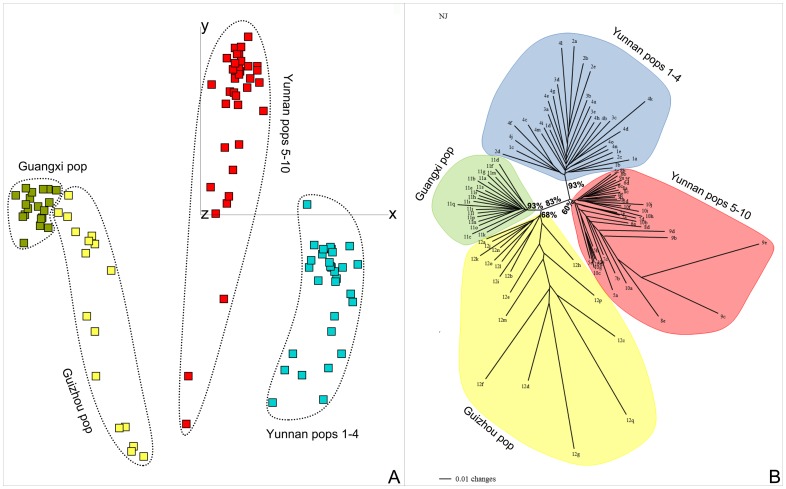
Four genetic lineages exist in *Paraisometrum mileense*. A. PCoA scatter plot based on AFLP data using the Jaccard distance on 12 populations of *P. mileense*. Axis 1(x) and 2(y). B. Unrooted NJ-tree based on Nei and Li’s restriction site distances. Bootstrap values only given for internal branches (1000 Neighbor-Joining replicates).

The PCoA (1^st^ axis 12% variance; 2^nd^ axis = 7.7%; 3^rd^ axis = 6.6%) separated the samples of the three regions strongly in the first axis, including the Yunnan populations 1–4 and 5–10 (Fig. 5A). Samples from Guangxi clustered very tightly together, while those from Guizhou and Yunnan were widely scattered. The samples from Guizhou were also scattered and almost overlapped with individuals from Guangxi at one end of their distribution, and with those from Yunnan, populations 5–10, at the other (Fig. 5A).

In the unrooted NJ tree, the samples clustered into four groups according to their province of origin including the split between Yunnan populations 1–4 and 5–10 (Fig. 5B). The Guangxi and Yunnan (populations 1–4) clusters received a high bootstrap support of 93%. These clusters were also characterized by short and uniform branch lengths. The same was the case for the cluster of Yunnan populations 5–10, except for three individuals, Yu8e, Yu9c and Yu9e. These had extremely long branches and were the same samples that seemed to be categorized as belonging to the Guizhou population in the STRUCTURE analysis (see Fig. 4C). The Guizhou population cluster was characterized by a loose clustering and relatively uniformly long branches (Fig. 5B).

## Discussion

### Taxonomic Status and Phylogenetic Affinities

The present study aimed to address several important issues surrounding this enigmatic, and until recently, monotypic genus that was thought to be extinct for 100 years. It was recently reported to occur only in one population in Shilin, Yunnan [4], but we were able to sample from two further populations, from two different provinces. Since all *Paraisometrum* samples in the phylogenetic study fell in a single strongly supported clade separated from other *Oreocharis* taxa by a long branch, they can be regarded as a single taxonomic unit (Figs. 3, 4). Unlike previous phylogenetic analysis where relationships of this genus to other congeners were unresolved [8], [12], [13], we found indications that the closest species to *Paraisometrum mileense* is *Ancylostemon hekouensis*, a recently described species endemic to the karst region in Southwest China [46], with a similar leaf and flower morphology to *Paraisometrum*, but without the distinct 4-lobed upper corolla lip. The species with the most similar corolla shape to *P. mileense*, *Ancylostemon saxatilis* Hemsl., was found not closely related to *P. mileense* (Figs. 3, 4). This morphological homoplasy, is in line with previous findings of high levels of parallelism in the evolution of floral morphology in *Oreocharis* [12].

### Phylogeographic History

Both, haplotype and nucleotype distribution, suggest a scenario of a close relationship between the Guangxi and Guizhou populations (shared nucleotypes), and a derived Yunnan population (lower haplo- and nucleotype diversity, derived nucleotypes) (Fig. 1, Table 3), and suggests a migration of *Paraisometrum* westward into Yunnan [9].

The significance in the Mantel test on the AFLP data indicates an isolation-by-distance scenario [47], and suggests a limited dispersal across the landscape. This may be a consequence of *Paraisometrum* being pollinated by bees, which have a relatively limited flying distance (e.g. [48], [49]), and the seeds of *Paraisometrum*, that, though small (ca. 0.6 mm long), are not dust-like, as in orchids [50], [51], to be dispersed far by wind, and do not have special dispersal aids, e.g. hooks or appendages (e.g. [52]) for long distance dispersal [53]. The significant genetic structure detected among the populations and regions (Tables 5, 6), further indicates a breakdown of their genetic connectivity. The ITS data may be used to give an indication of the divergence time between the *Paraisometrum* localities. Taking the average rate of evolution of the ITS spacers of 11 herbaceous plants (4.13×10^−9^ substitutions per site per year) [54], [55], the Yunnan populations might have separated from the other populations around 1.6 million years ago (±0.26 SE), at the beginning of the Pleistocene. This is a period that would cover repeated glacial-interglacial cycles during which limited secondary contact may have occurred (see below). This could explain the higher levels of within-population genetic variation observed here.

Our study provides a first insight into the history of a plant considered extinct in the wild. To fully address the evolution of the species, and to untangle historic from contemporary events, an increased sampling is required, additional markers employed such as microsatellites, combined with population demographic analyses (e.g. [56], [57]), and, with view to conservation, ecological niche modelling (e.g. [58]).

### Genetic Diversity and Differentiation

The STRUCTURE analysis indicated several noteworthy aspects of the *Paraisometrum* populations; firstly, that the Yunnan populations represented two distinct lineages with at least three gene pools and can be divided into two main populations, populations 1–4, and populations 5–10 with some admixture (Fig. 4). This bipartition of the Yunnan populations was also seen in the PCoA clustering analysis (Figs. 5A, S5) and the NJ tree (Fig. 5B). Both groups have the same haplotypes which indicates their common ancestry, and the same composition of ITS nucleotypes (Table 3), which suggests fragmentation of a previously more continuously distributed population. Populations 1–4 occur near villages, in the foothills of Mt. Guishan in secondary open forests with frequent disturbance due to human activities, while populations 5–10 grow mostly undisturbed further away from villages, above the foothills almost to the summit of Mt. Guishan, and in more dense and primary forests (Fig. 2B, Table 1). Whether the bipartition is linked to the condition of the habitats surrounding the population groups, being disconnected from each other by disturbed forest, or has an older origin, would require further research. Secondly, the population in Guangxi was distinct and very homogeneous, also seen in the tight clustering in the PCoA (Fig. 5A) and the uniformly short branches in NJ tree (Fig. 5B). This might indicate that while the plants contain similar genetic diversity levels as other regions, the plants were closer related to each other possibly due to consanguineous matings, perhaps as a side effect of their small population sizes and limited area of distribution of less than a hectare (Table 1), and long distance to the other distribution localities with no apparent intervening populations known (Fig. 1). Thirdly, on the contrary, the internal and terminal branches of the population from Guizhou were long, and the STRUCTURE analysis suggested that some plants had a genetic makeup greatly similar to plants of the Yunnan populations 8 (plant Yu8e) and 9 in particular (plants Yu9c, Yu9e). This was not reflected in a mixed clustering of the respective Yunnan samples among the Guizhou samples, though their branches were unusually long (Fig. 5B). This might reflect genetic links and allele exchanges between these populations, perhaps due to habitat expansion during glacial-interglacial cycles that brought the populations in closer proximity (e.g. [58]). They are currently only some 120 km apart (Fig. 1).

The genetic diversity found at the population level was very low, although this might have been a consequence of the low number of individuals (often 5) included for analysis of the Yunnan populations. However, even when calculated within the three regions, the AFLP diversity levels were relatively low, with no marked difference between the regions (Table 4). Even at the species level, the genetic diversity of *Paraisometrum* was comparatively low (*He* = 0.144), and much lower compared to *Primulina tabacum* (*He* = 0.339), another herbaceous Gesneriaceae species from China [11]. *Primulina tabacum* and *P. mileense* have similar distribution sizes though in different provinces (*P. tabacum* in Guangdong and Hunan, *Paraisometrum* in Guangxi, Guizhou and Yunnan), but both occur in limestone karst areas, though at different altitudes (*P. tabacum* below 300 m, *Paraisometrum* between 1,180–2,500 m) and in different aspects (*P. tabacum* grows around entrances of limestone caves, *Paraisometrum* in limestone forests). The high genetic diversity levels found in *P. tabacum* populations were explained by their refugial history and/or breeding system [11]. The more restricted habitats in cave entrances of *P. tabacum*, as opposed to the more open forest habitats of *Paraisometrum* could be a factor that may have allowed a more continuous distribution and genetic connectivity of *Paraisometrum* populations. However, it is more likely that the greater population sizes of *P. tabacum* (1,000 individuals per population, as opposed to 30–150 individuals in *Paraisometrum* populations) has allowed *P. tabacum* to retain higher diversity levels. The similarly low levels of AFLP diversity compared to *Paraisometrum* found in other herbaceous perennials, such as *Trollius europaeus* L. (*He* = 0.158–0.229) [24], *Silene otitis* St.-Lag. (*He* = 0.167–0.240) [25], and *Draba aizoides* L. (*He* = 0.07–0.15) [26], were linked to effects of population fragmentation. Thus, the low genetic diversity in *Paraisometrum* is likely a result of a combination of fragmentation and small population size. Intriguingly though, in *Draba* L., the low genetic diversity levels were not correlated with a limited reproductive fitness, as suggested by their high germination rates. This may have important conservation implications and could be a field of study in *Paraisometrum* in the future.

### Implications for Conservation

Our work provides an example of an integrated study for endemic species in the karst regions in South China with extremely small populations. It is well known that the karst region is characterized by a limestone topography with an abundance of endemic plants with extremely small populations [7], [59], [60]. In these regions, localised endemics occur often restricted to one or few limestone hills, which are usually isolated by non-limestone topography and the plants effectively occur on islands with high risks of extinction [7], [14]. The three distribution points of *Paraisometrum* are isolated in different limestone forests, and the occurrence nearby villages is not unusual for endemic plants of the karst region [59], [61].

The villages are commonly closely associated with characteristic forests on the limestone hills, the Feng Shui Forests. These are important for the water supply of the villages, but are recently influenced by activities of local people, such as free range poultry and goat keeping [62], which negatively affect the forest habitats.

With a view to conservation of *Paraisometrum*, the only three distribution points known to date appear to contain a significant amount of diversity within their populations with only a limited level of differentiation. Though, analysing a larger number of individuals per population might show some more resolution. Furthermore, the STRUCTURE analysis shows that they seem to have experienced some gene flow between Yunnan and Guizhou populations and are thus not genetically greatly isolated (Table 1). However, the calculated levels of genetic diversity were quite low, even when estimated across the regions including Yunnan (and not based on the small ‘sub-populations’ there). This is likely linked to the relatively small population sizes. The low genetic diversity might hinder adaptation to rapid climatic changes, as shown in the exceptionally dry season of 2011 during which they contracted significantly, due to the death of many immature individuals, and hardly any flower was produced in the Yunnan populations, their inflorescences withered (SYM, pers. observation). The relatively uniform distribution of genetic diversity in the three provinces of *Paraisometrum* does not immediately allow a prioritization of regions or populations for conservation efforts. Even though little differences in the genetic make-up were detected between the two Yunnan lineages, the one including populations 1–4, may deserve stronger protection due to their close proximity to human habitation and the negative effects these can bring (Fig. 2). For all of the reasons given above, conservation efforts should thus include all populations and include *in situ* as well as *ex situ* measures to safeguard this enigmatic species.

*In situ* measures relevant to *Paraisometrum* have already been implemented in Yunnan on a small scale at Shilin populations and involved the removal of *Ageratina adenophora* (Spreng.) R. M. King & H. Robinson, an invasive plant alien to China [63], the exclusion of goats through fencing in the plants, and by involving the local villagers to safeguard these plants that have a high potential for eco-tourism. Future *in situ* measures may also involve forest restoration [64], particularly for populations near villages. However, these may not be sufficient and *ex situ* conservation measures are required alongside *in situ* measures in the light of the observed strong detrimental effects of recent rainfall variation on the populations. This may, for the moment preclude transfer of plants from threatened habitats to new localities in the wild. Though, the plants are easily cultivated and propagated vegetatively and through seeds. Currently, about 56 plants are cultivated at the Kunming Botanical Garden, Yunnan, China, and seeds of three populations have been deposited at the Germplasm Bank of Wild Species, Plant Germplasm and Genomics Center, Kunming Institute of Botany, Yunnan, China. Thus, the immediate and medium term survival of the species seems ensured. However, seeds in germplasm banks are prone to genetic erosion during seed rejuvenation cycles even of inbreeding species [65]. Currently research is underway studying the pollinators to elucidate the reproductive strategy and breeding system to better devise a tailored *ex situ* strategy for *Paraisometrum mileense.*

## Conclusions

The erstwhile monotypic genus *Paraisometrum mileense* (now *Oreocharis mileensis*) represents an isolated and independent taxonomic unit, and appears closest related to the erstwhile *Ancylostemon hekouensis* (now *Oreocharis hekouensis*). The populations in Yunnan seem to have derived from those in Guangxi and Guizhou with some evidence of limited gene transfer between Yunnan and Guizhou. The occurrence points in Yunnan were divided into two quite distinct lineages, while the population in Guangxi is relatively homogeneous. Overall, the populations of *P. mileense* contain relatively low levels of genetic diversity with no apparent gradient across the species’ range. Several conservation measures are currently implemented, but additional actions are needed. From a conservation genetic perspective, all populations seem equally important for *in situ* and *ex situ* protection.

## Supporting Information

Figure S1 **Comparison of posterior probability values between run 1 versus run 2 (10% burn-in) of the Bayesian inference analysis.**
(TIF)

Figure S2 **Posterior probabilities of splits at selected increments over MCMC run1 (A) and 2 (B) of the Bayesian inference analysis.**
(TIF)

Figure S3 **Comparisons of topological differences within and among MCMC runs of the Bayesian inference analysis.**
(TIF)

Figure S4 **Maximum parsimony strict consensus tree of 12 parsimonious trees of 1538 steps, based on combined ITS and** ***trn*** **LF sequence data (CI = 0.5566; RI = 0.6982).** Numbers above branches are bootstrap values (x denotes branches receiving <50% support).
(TIF)

Figure S5 **PCoA scatter plot based on AFLP data using the Jaccard distance on 12 populations of Paraisometrum mileense. Axes 1 (x) and 3 (z).**
(TIF)

Table S1 **Collection details and GenBank numbers of** ***Oreocharis*** **(including** ***Paraisometrum mileense*** **) and outgroup samples used for the phylogenetic analyses.**
(DOC)

Table S2 **Characteristics of the Bayesian inference analysis of 86 samples of combined ITS and** ***trn*** **LF data.**
(DOC)

Table S3 **Binary AFLP data for 12 populations of Paraisometrum mileense from Yunnan (Yu), Guizhou (Gz) and Guangxi (GX).**
(XLSX)

## References

[1] Smith L (2007) ‘Extinct’ plant found flowering in China. The Times website. Available: http://www.thetimes.co.uk/tto/news/world/article1973186.ece. Accessed 2014 Sep 1.

[2] Weitzman AL, Skog LE, Wang WT, Pan KY, Li ZY (1997) New taxa, new combinations, and notes on Chinese Gesneriaceae. Novon 7: 423–435.

[3] Shui YM, Cai J (2007) “100-years-lost” plant reemerges in Yunnan, China. Samara 12: 3.

[4] Ma YP, Chen G, Grumbine RE, Dao ZL, Sun WB, et al (2013) Conserving plant species with extremely small populations (PSESP) in China. Biodivers Conserv 22: 803–809.

[5] Xu WB, Pan B, Huang YS, Ye XX, Liu Y (2009) *Paraisometrum* W.T.Wang, a newly recorded genus of Gesneriaceae from Guangxi, China. Guihaia 29: 581–583.

[6] Gao Q, Xu WB (2011) *Paraisometrum* W. T. Wang, a newly recorded genus of Gesneriaceae from Guizhou, China. Acta Bot Boreal-Occid Sin 31 858–860.

[7] Wei YG, Wen F, Möller M, Monro A, Zhang Q, et al.. (2010) Gesneriaceae of South China. Nanning: Guangxi Science and Technology Publishing House. 777 p.

[8] Möller M, Forrest A, Wei YG, Weber A (2011) A molecular phylogenetic assessment of the advanced Asiatic and Malesian didymocarpoid Gesneriaceae with focus on non-monophyletic and monotypic genera. Plant Syst Evol 292(3–4): 223–248.

[9] Lowe A, Harris S, Ashton P (2007) Chapter 3. Genetic diversity and differentiation. In: Ecological Genetics: Design, Analysis, and Application. Hongkong: Blackwell Publishing. pp. 52–100.

[10] Ni XW, Huang YL, Wu L, Zhou RC, Deng SL, et al (2006) Genetic diversity of the endangered Chinese endemic herb *Primulina tabacum* (Gesneriaceae) revealed by amplified fragment length polymorphism (AFLP). Genetica 127: 177–183. 16850222 10.1007/s10709-005-3227-0

[11] Wang CN, Möller M, Cronk QCB (2004) Population genetic structure of *Titanotrichum oldhamii* (Gesneriaceae), a subtropical bulbiliferous plant with mixed sexual and asexual reproduction. Ann Bot 93: 201–209. 14707003 10.1093/aob/mch028 PMC4241082

[12] Möller M, Middleton DJ, Nishii K, Wei YG, Sontag S, et al (2011) A new delineation for *Oreocharis* incorporating an additional ten genera of Chinese Gesneriaceae. Phytotaxa 23: 1–36.

[13] Tan Y, Wang Z, Sui XY, Hu GW, Motley T, et al (2011) The systematic placement of the monotypic genus *Paraisometrum* (Gesneriaceae) based on molecular and cytological data. Pl Diversity Resources 33: 465–476.

[14] Li ZY, Wang YZ (2004) Plants of Gesneriaceae in China. Zhengzhou: Henan Science and Technology Publishing House. 14–79 p.

[15] Pan KY (1987) Taxonomy of the genus *Oreocharis* (Gesneriaceae). Acta Phytotax Sin 25: 164–293.

[16] Wang WT, Pan KY, Li ZY (1990) Gesneriaceae. In: Wang WT, editor. Fl. Reipubl. Popularis Sin. 69: 190–203. Science Press, Beijing.

[17] Wang WT, Pan KY, Li ZY, Weitzman AL, Skog LE (1998) Gesneriaceae. In: Wu ZY, Raven PH (eds) Flora of China. 18: 268–272. Beijing: Science Press & St Louis: Missouri Botanical Garden Press.

[18] Jiao LJ, Shui YM (2013) Evaluating candidate DNA barcodes among Chinese *Begonia* (Begoniaceae) species. Pl Diversity Resources 35: 715–724.

[19] Liu J, Möller M, Gao LM, Zhang DQ, Li DZ (2011) DNA barcoding for the discrimination of Eurasian yews (*Taxus* L., Taxaceae) and the discovery of cryptic species. Mol Ecol Res 11: 89–100. 10.1111/j.1755-0998.2010.02907.x 21429104

[20] Liu ML, Yu WB, Wang H (2013) Rapid identification of plant species and iflora: application of DNA barcoding in a large temperate genus *Pedicularis* (Orobanchaceae). Pl Diversity Resources 35: 707–714.

[21] Ren H, Peng SL, Zhang JX, Jian SG, Wei Q, et al (2003) The ecological and biological characteristics of an endangered plant, *Primulina tabacum* Hance. Acta Ecol Sin 23: 1012–1017.

[22] Rafalski JA, Vogel JM, Morgante M, Powell W, Andre C, et al.. (1997) Generating and using DNA makers in plants. In: Birren B, Lai E, editors. Non-mammalian Genome Analysis: A practical Guide. New York: Academic Press, pp. 75–134.

[23] Vos P, Hogers R, Bleeker M, Reijans M, van de Lee T, et al (1995) AFLP: a new technique for DNA fingerprinting. Nucleic Acids Res 23: 4407–4414. 7501463 10.1093/nar/23.21.4407 PMC307397

[24] Despres L, Loriot S, Gaudeul M (2002) Geographic pattern of genetic variation in the European globeflower *Trollius europaeus* L. (Ranunculaceae) inferred from amplified fragment length polymorphism markers. Mol Ecol 11: 2337–2347. 12406244 10.1046/j.1365-294x.2002.01618.x

[25] Lauterbach D, Ristow M, Gemeinholzer B (2012) Population genetics and fitness in fragmented populations of the dioecious and endangered *Silene otites* (Caryophyllaceae) Plant Syst Evol. 298: 155–164.

[26] Vogler F, Reisch C (2013) Vital survivors: low genetic variation but high germination in glacial relict populations of the typical rock plant *Draba aizoides* . Biodivers Conserv 22: 1301–1316.

[27] Bonin A, Ehrich D, Manel S (2007) Statistical analysis of amplified fragment length polymorphism data: a toolbox for molecular ecologists and evolutionists. Mol Ecol 16: 3737–3758. 17850542 10.1111/j.1365-294X.2007.03435.x

[28] Weber A, Clark JL, Möller M (2013) A new formal classification of Gesneriaceae. Selbyana 31(2): 68–94.

[29] Doyle JJ, Doyle JL (1987) A rapid DNA isolation procedure for small quantities of fresh leaf tissue. Phytochem Bull 19: 11–15.

[30] Doyle JJ, Doyle JL (1990) Isolation of plant DNA from fresh tissue. Focus 12: 13–15.

[31] White TL, Bruns T, Lee S, Taylor J (1990) Amplification and direct sequencing of fungal ribosomal RNA genes for phylogenetics. In: Innis MA, Gelfand D, Sninsky JJ, White TJ, editors. PCR protocols: a guide to methods and applications. San Diego: Academic Press, pp. 315–322.

[32] Taberlet P, Gielly L, Pautou G, Bouvet J (1991) Universal primers for amplification of three non-coding regions of chloroplast DNA. Plant Mol Biol 17: 1105–1109. 1932684 10.1007/BF00037152

[33] Bonin A, Bellemain E, Bronken Eidesen P, Pompanoni F, Brochmann C, et al (2004) How to track and assess genotyping errors in population genetics studies. Mol Ecol 13: 3261–3273. 15487987 10.1111/j.1365-294X.2004.02346.x

[34] Weber A, Wei YG, Puglisi C, Wen F, Mayer V, et al (2011) A new definition of the genus *Petrocodon* (Gesneriaceae). Phytotaxa 23: 49–67.

[35] Swofford DL (2002) PAUP*: phylogenetic analysis using parsimony (*and other methods), version 4. Sinauer, Sunderland, Massachusetts, USA.

[36] Huelsenbeck JP, Ronquist F (2001) MRBAYES: Bayesian inference of phylogenetic trees. Bioinformatics 17: 754–755. 11524383 10.1093/bioinformatics/17.8.754

[37] Ronquist F, Teslenko M, van der Mark P, Ayres D, Darling A, et al (2011) MrBayes 3.2: Efficient Bayesian phylogenetic inference and model choice across a large model space. Syst Biol 61: 539–542. 10.1093/sysbio/sys029 PMC3329765 22357727

[38] Farris J, Källersjö SM, Kluge AG, Bult C (1994) Constructing a significance test for incongruence. Syst Biol 44: 570–572.

[39] Farris J, Källersjö SM, Kluge AG, Bult C (1995) Testing significance of incongruence. Cladistics 10: 315–319.

[40] Nylander JA, Wilgenbusch JC, Warren DL, Swofford DL (2008) AWTY (are we there yet?): a system for graphical exploration of MCMC convergence in Bayesian phylogenetics. Bioinformatics 24(4): 581–583. 17766271 10.1093/bioinformatics/btm388

[41] Peakall R, Smouse PE (2006) GenAlEx 6.5: genetic analysis in Excel. Population genetic software for teaching and research-an update. Mol Ecol Notes 6: 288–295. 10.1093/bioinformatics/bts460 PMC3463245 22820204

[42] Pritchard JK, Stephens M, Donnelly P (2000) Inference of population structure using multilocus genotype data. Genetics 155: 945–959. 10835412 10.1093/genetics/155.2.945 PMC1461096

[43] Falush D, Stephens M, Pritchard JK (2003) Inference of population structure using multilocus genotype data: linked loci and correlated allele frequencies. Genetics 164: 1567–1587. 12930761 10.1093/genetics/164.4.1567 PMC1462648

[44] Evanno G, Regnaut S, Goudet J (2005) Detecting the number of clusters of individuals using the software STRUCTURE: a simulation study. Mol Ecol 14: 2611–2620. 15969739 10.1111/j.1365-294X.2005.02553.x

[45] Casgrain P, Legendre P, Vaudor A (2005) The royal package for multivariate and spatial analysis, Version 4.0 (development release 10). Available: http://adn.biol.umontreal.ca/~numericalecology/old/R/index.html. Accessed 2014 Sep 1.

[46] Chen WH, Shui YM (2006) *Ancylostemon hekouensis* (Gesneriaceae), a new species from Yunnan, China. Ann Bot Fenn 43: 448–450.

[47] Wright S (1943) Isolation by distance. Genetics 28: 114–138. 17247074 10.1093/genetics/28.2.114 PMC1209196

[48] Pasquet RS, Peltier A, Hufford MB, Oudin E, Saulnier J, et al (2008) Long-distance pollen flow assessment through evaluation of pollinator foraging range suggests transgene escape distances. P Natl Acad Sci USA 105(36): 13456–13461. 10.1073/pnas.0806040105 PMC2533211 18768793

[49] Eckert JE (1933) The flight range of the honeybee. J Agric Res 47: 257–285.

[50] Dressler RL (1993) Phylogeny and classification of the orchid family. Cambridge: Cambridge University Press, 314 p.

[51] Murren CJ, Ellison AM (1998) Seed dispersal characteristics of *Brassavola nodosa* (Orchidaceae). Am J Bot 85(5): 675–680. 21684949

[52] Kokubugata G, HirayamaY, Peng CI, Yokota M, Möller M (2011) Phytogeographic aspects of *Lysionotus pauciflorus* sensu lato (Gesneriaceae) in the China, Japan and Taiwan regions: phylogenetic and morphological relationships and taxonomic consequences. Plant Syst Evol 292(3–4): 177–188.

[53] Chen WH (2013) Reproductive Biology of *Paraisometrum* W. T. Wang, an endemic to SW China– with special emphasis on its phylogenetic position and genetic diversity. Ph.D. Thesis, Kunming Institute of Botany, Chinese Academy of Sciences, Kunming, China.

[54] Kay KM, Whittall JB, Hodges SA (2006) A survey of nuclear ribosomal internal transcribed spacer substitution rates across angiosperms: an appropriate molecular clock with life history effects. BMC Evol Biol 6: 36. 16638138 10.1186/1471-2148-6-36 PMC1484492

[55] Puglisi C, Wei YG, Nishii K, Möller M (2011) *Oreocharis* × *heterandra* (Gesneriaceae): a natural hybrid from the Shengtangshan Mountains, Guangxi, China. Phytotaxa 38: 1–18.

[56] Ribeiro RA, Lemos-Filho JP, Ramos ACS, Lovato MB (2011) Phylogeography of the endangered rosewood *Dalbergia nigra* (Fabaceae): insights into the evolutionary history and conservation of the Brazilian Atlantic Forest. Heredity 106: 46–57. 20517347 10.1038/hdy.2010.64 PMC3183853

[57] Liu J, Möller M, Provan J, Gao LM, Poudel RC, et al (2013) Geological and ecological factors drive cryptic speciation of yews in a biodiversity hotspot. New Phytol 199: 1093–1108. 23718262 10.1111/nph.12336

[58] Poudel RC, Möller M, Gao LM, Ahrends A, Baral SR, et al (2012) Using morphological, molecular and climatic data to delimitate yews along the Hindu Kush-Himalaya and adjacent regions. PLoS ONE 7(10): e46873. 23056501 10.1371/journal.pone.0046873 PMC3466193

[59] Fang RZ, Bai PY, Huang GB, Wei YG (1995) A floristic study on the seed plants from tropics and subtropics of Dian-Qian-Gui. Acta Bot Yunnan Suppl. 5: 111–150.

[60] Sun H (2013) Phytogeographical regions of China. In: Hong DY, Blackmore S, editors. Plants of China: A companion to the Flora of China. Beijing: Sciences Press. pp. 176–204.

[61] Chen WH, Möller M, Shui YM, Wang H, Wen F, et al (2014) Three new species of *Petrocodon* (Gesneriaceae), endemic to the limestone areas of Southwest China, and preliminary insights into the diversification patterns of the genus. Syst Bot 39(1): 316–330.

[62] Xu HG, Zhou F (2011) Biodiversity in the karst area of Southwest Guangxi. Beijing: Encyclopedia of China Publishing House. 143 p.

[63] Zhu L, Sun OJ, Sang WG, Li ZY, Ma KP (2007) Predicting the spatial distribution of an invasive plant species (*Eupatorium adenophorum*) in China. Landscape Ecol 22(8): 1143–1154.

[64] Lamb D, Erskine PD, Parrotta JA (2005) Restoration of degraded tropical forest landscapes. Science 310 (5754): 1628–1632. 10.1126/science.1111773 16339437

[65] Parzies HK, Spoor W, Ennos RA (2000) Genetic diversity of barley landrace accessions (*Hordeum vulgare* ssp. *vulgare*) conserved for different lengths of time in *ex situ* gene banks. Heredity 84: 476–486. 10849072 10.1046/j.1365-2540.2000.00705.x

