# Supplementary figures and images for: Taxonomic Status, Phylogenetic Affinities and Genetic Diversity of a Presumed Extinct Genus, Paraisometrum W.T. Wang (Gesneriaceae) from the Karst Regions of Southwest China
Source: PLoS One. 2014 Sep 24;9(9):e107967. doi: 10.1371/journal.pone.0107967 (PMC4176718; doi:10.1371/journal.pone.0107967)

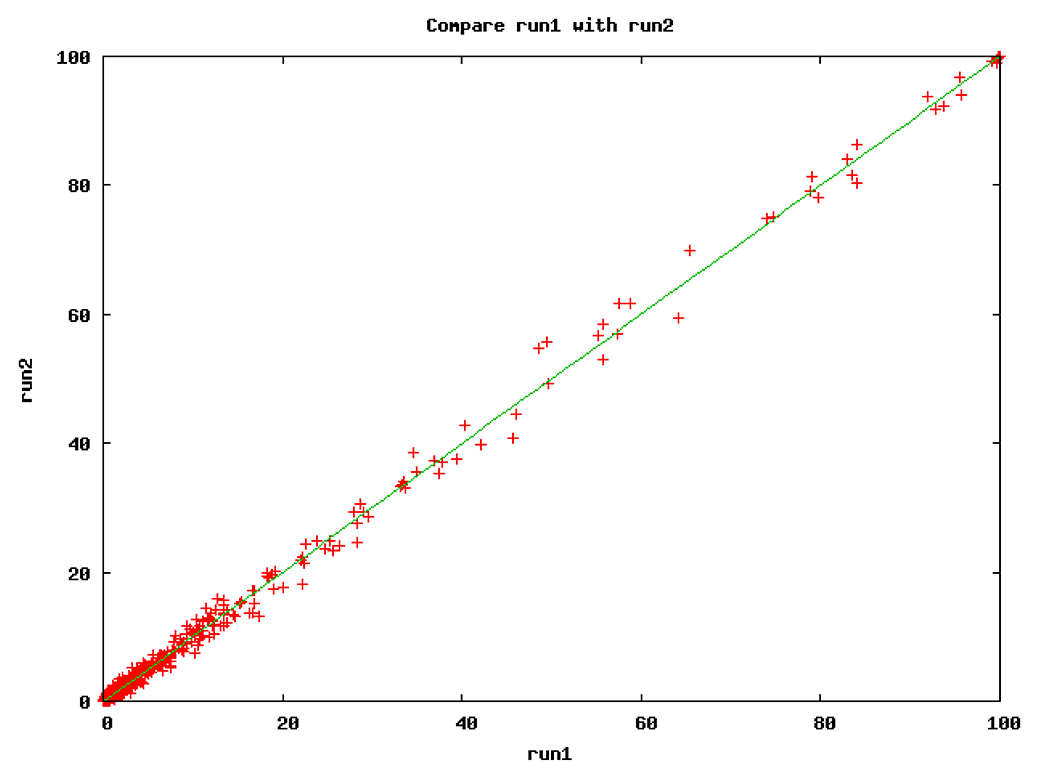

Supplement: Figure S1 — Comparison of posterior probability values between run 1 versus run 2 (10% burn-in) of the Bayesian inference analysis. (TIF) [file pone.0107967.s001.tif]

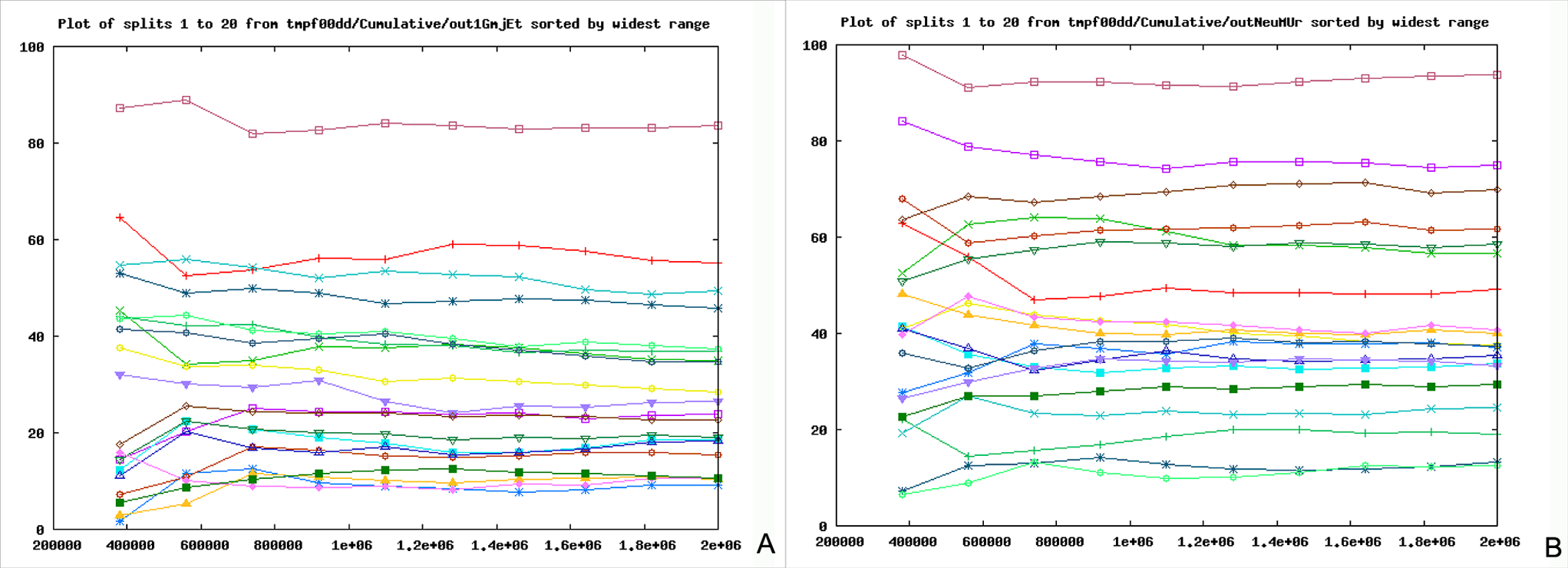

Supplement: Figure S2 — Posterior probabilities of splits at selected increments over MCMC run1 (A) and 2 (B) of the Bayesian inference analysis. (TIF) [file pone.0107967.s002.tif]

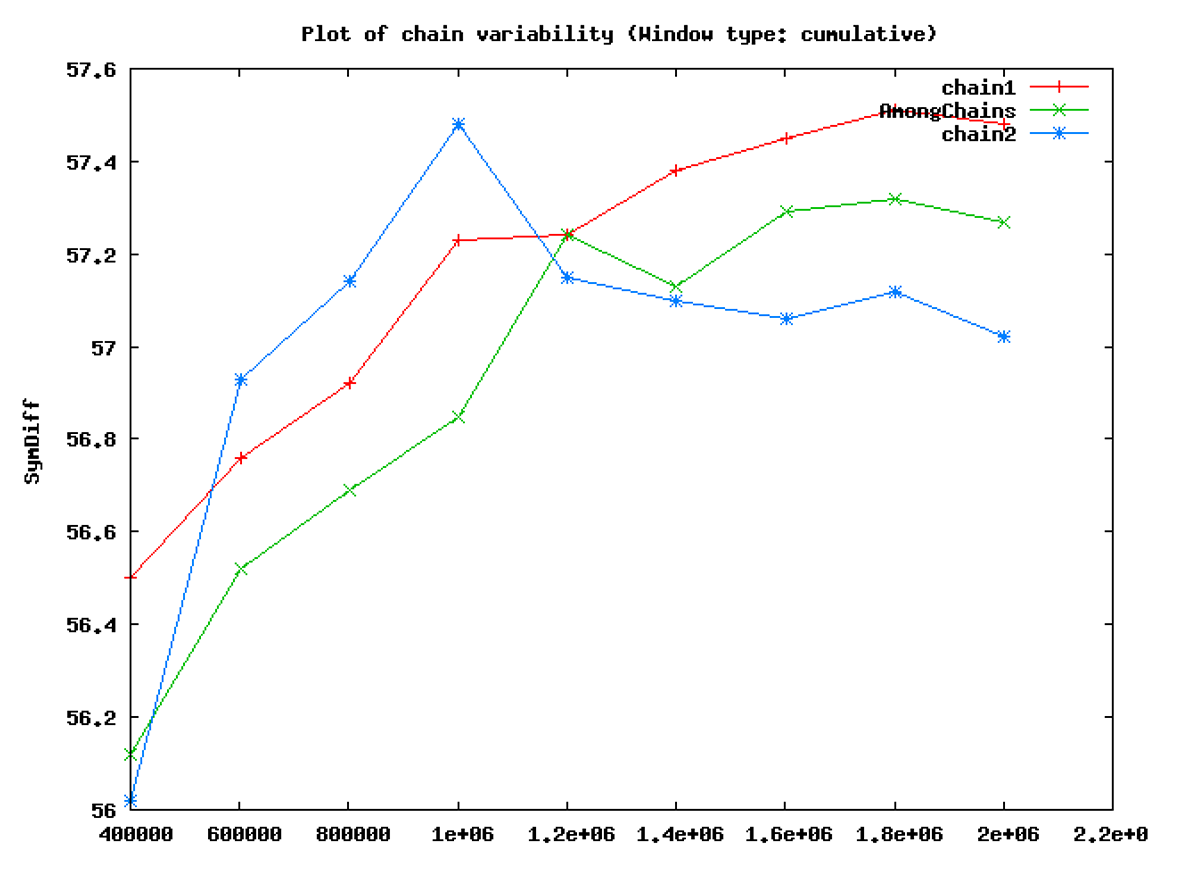

Supplement: Figure S3 — Comparisons of topological differences within and among MCMC runs of the Bayesian inference analysis. (TIF) [file pone.0107967.s003.tif]

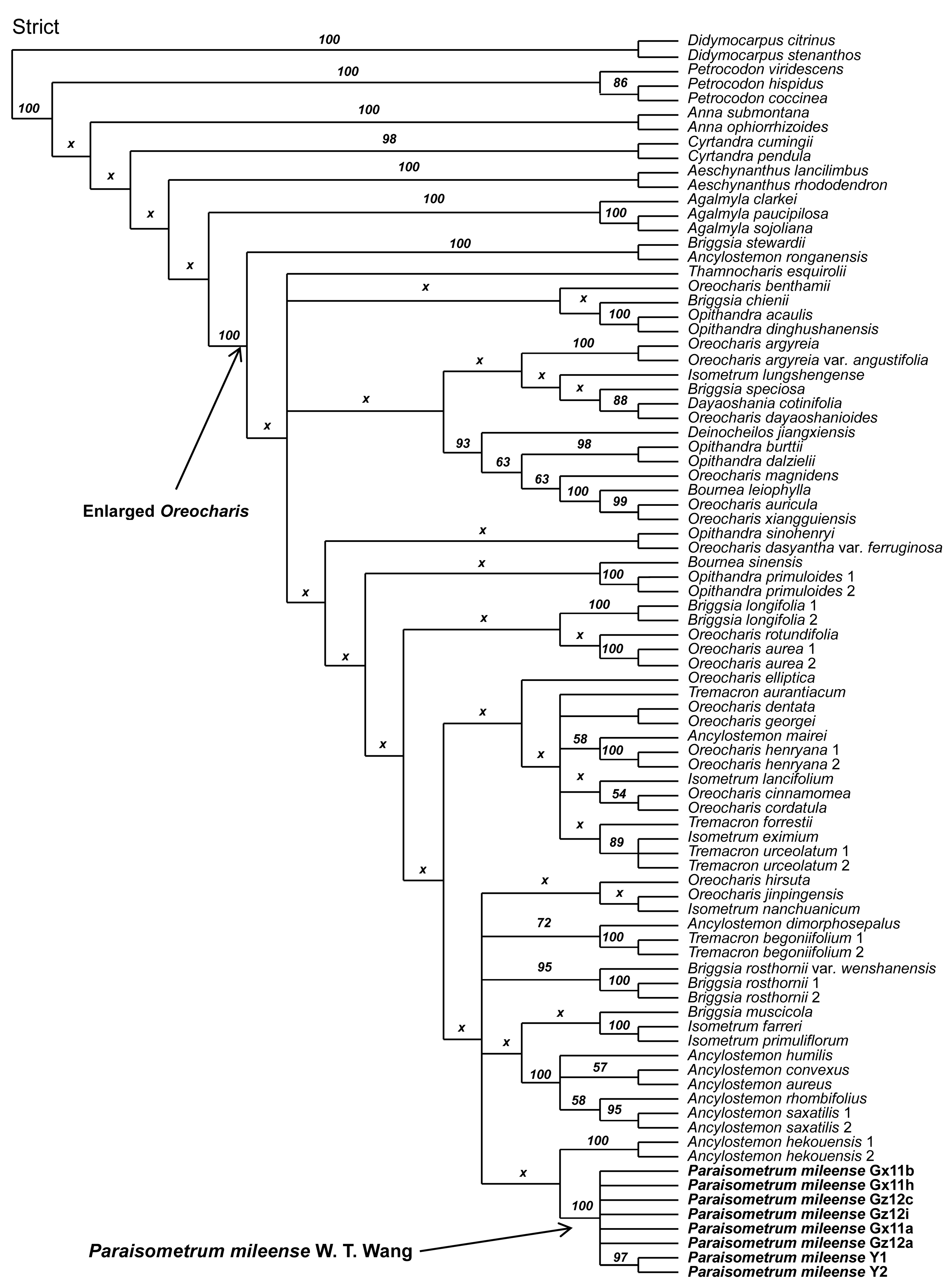

Supplement: Figure S4 — Maximum parsimony strict consensus tree of 12 parsimonious trees of 1538 steps, based on combined ITS and trn LF sequence data (CI = 0.5566; RI = 0.6982). Numbers above branches are bootstrap values (x denotes branches receiving <50% support). (TIF) [file pone.0107967.s004.tif]

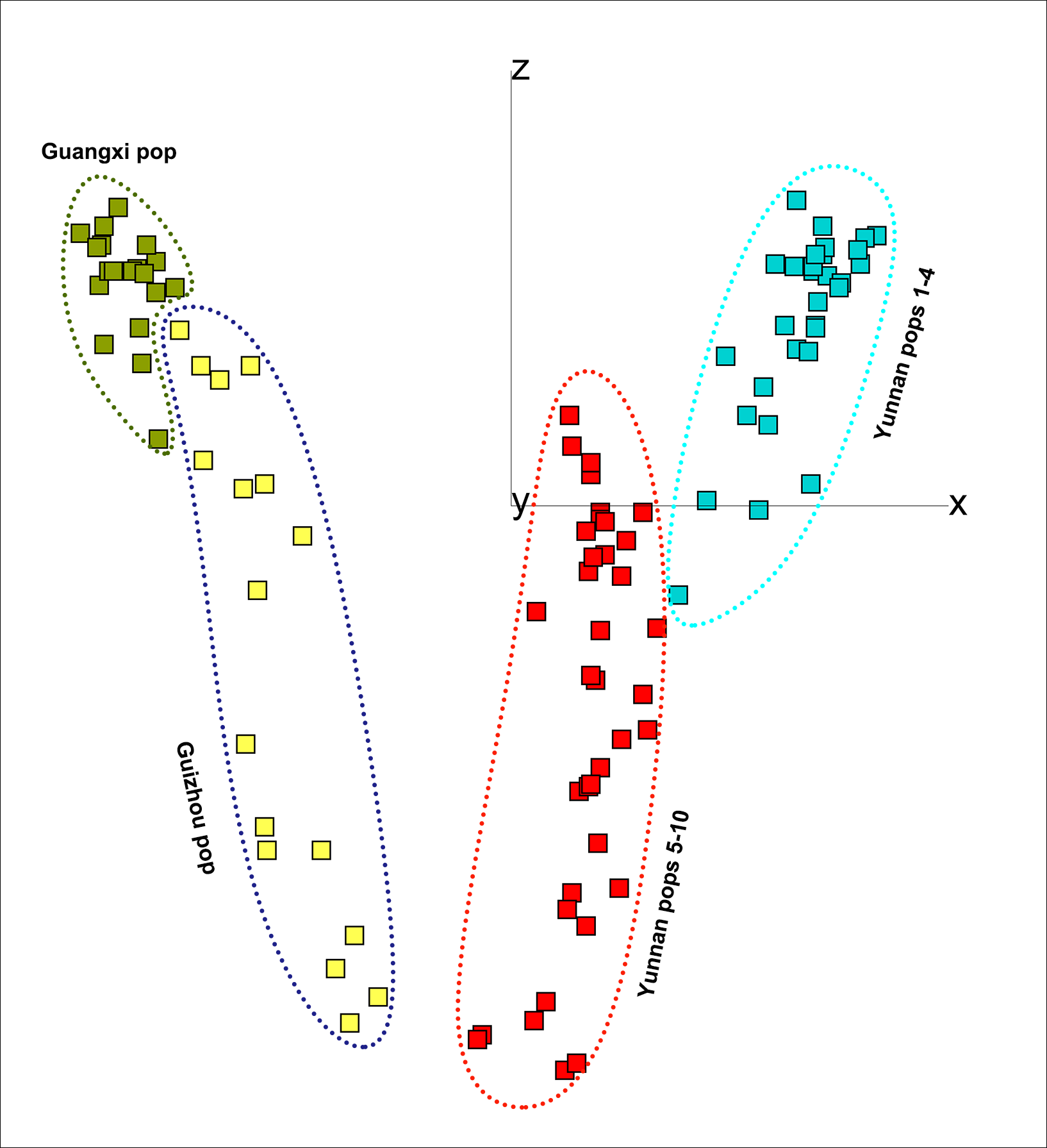

Supplement: Figure S5 — PCoA scatter plot based on AFLP data using the Jaccard distance on 12 populations of Paraisometrum mileense. Axes 1 (x) and 3 (z). (TIF) [file pone.0107967.s005.tif]
