# Supplementary material for: Taxonomic Status, Phylogenetic Affinities and Genetic Diversity of a Presumed Extinct Genus, Paraisometrum W.T. Wang (Gesneriaceae) from the Karst Regions of Southwest China
Source: PLoS One. 2014 Sep 24;9(9):e107967. doi: 10.1371/journal.pone.0107967 (PMC4176718; doi:10.1371/journal.pone.0107967)
Supplement: Table S1 — Collection details and GenBank numbers of Oreocharis (including Paraisometrum mileense ) and outgroup samples used for the phylogenetic analyses. (DOC) [file pone.0107967.s006.doc]

**Supporting Information**

**Table S1 Collection details and GenBank numbers of *Oreocharis* (including *Paraisometrum mileense*) and outgroup samples used for the phylogenetic analyses.**

| Taxon | Current name | Location | Voucher specimens | Deposited in | *trn*L-F | ITS or ITS1 / ITS2 |
| --- | --- | --- | --- | --- | --- | --- |
| **Outgroup samples:** |  |  |  |  |  |  |
| *Aeschynanthus lancilimbus* W.T.Wang | - | China: unknown locality | Y.Z. Wang S-10868 | PE | FJ501499 | HQ632992 |
| *Aeschynanthus rhododendron* Ridley | - | Peninsular Malaysia, Genting Highlands | P. Woods 600 [cult. RBGE 19680624] | E | HQ632895 | FJ501333 |
| *Agalmyla clarkei* (Elmer) B.L.Burtt | - | Philippines: Luzon, Barangay Penicuason | RBGE-PNH1997 IS26 [cult. RBGE 19972530A] | E | _ | FJ501360 |
| *Agalmyla clarkei* (Elmer) B.L.Burtt | - | Philippines: Leyte Island, Mt. Lobi | RBGE-PNH1999 (P99) 13 [cult. RBGE 19991911] | E | FJ501540 | _ |
| *Agalmyla paucipilosa* Hilliard & B.L.Burtt | - | Indonesia: Sulawesi, Mt. Rantemario | Smith & Galloway 261 | E | HQ632893 | HQ632990 |
| *Agalmyla sojoliana* Hilliard & B.L.Burtt | - | Indonesia: Sulawesi, Mt. Sojol | Smith & Galloway 321 | E | HQ632894 | HQ632991 |
| *Anna ophiorrhizoides* (Hemsl.) B.L.Burtt & Davidson | - | China: Sichuan, Mt. Emei | Y. M. Shui et al. B2012-058 | KUN | This study | This study |
| *Anna submontana* Pellegr. | - | China:Yunnan, Hekou county | Y.M. Shui et al. 94841 | KUN | This study | This study |
| *Cyrtandra cumingii* C.B.Clarke | - | Japan: Ruykyus, Iriomote Island | G. Kokubugata 11134 | TNS | HQ632905 | HQ633002 |
| *Cyrtandra pendula* Blume | - | Peninsular Malaysia, Negeri Sembilan, Kuala Pilah distr., Jeram Toi | A. Weber & Anthonysamy 860730-1/2 [cult. HBV] | WU | FJ501530 | FJ501354 |
| *Didymocarpus citrinus* Ridley | - | Peninsular Malaysia, Perlis, Kedah Peak | P. Davis 69437 [cult. RBGE 19830510] | E | AJ492293 | DQ912669 |
| *Didymocarpus stenanthos* C.B.Clarke | - | China: Yunnan, Binchuan county | M. Möller et al. MMO 01-156 | E, WU | FJ501512 | DQ91268 |
| *Petrocodon coccineus* (C.Y.Wu ex H.W.Li) Yin.Z.Wang | - | China: Yunnan, Malipo county | GBOWS 290 | KUN | KF202299 | KF202292 |
| *Petrocodon hispidus* (W.T.Wang) A.Weber & Mich.Möller | - | China: Yunnan, Xichou county | Y.M. Shui et al. B2012-082 | KUN | KF202300 | KF202293 |
| *Petrocodon viridescens* W.H.Chen, Mich.Möller & Y.M..Shui | - | China: Yunnan, Maguan county | Y.M. Shui et al. 85339 | KUN | KF202304, | KF202297 |
|  |  |  |  |  |  |  |
| **Ingroup samples:** |  |  |  |  |  |  |
| *Ancylostemon aureus* (Franch.) B.L.Burtt | *Oreocharis* *concava* (Craib) Mich.Möller & A.Weber | China: Yunnan, Binchuan county | Y.M. Shui et al. B426 | KUN | This study | This study |
| *Ancylostemon convexus* Craib | *O.* *convexa* (Craib) Mich.Möller & A.Weber | China: Yunnan, Dali county | M. Möller et al. MMO 01-176 | E, WU | FJ501506 | FJ501337 |
| *Ancylostemon dimorphosepalus* W.H.Chen & Y.M.Shui | - | China: Yunnan, Yuanyang county | Y.M. Shui et al. 85333 | KUN | This study | This study |
| *Ancylostemon hekouensis* Y.M.Shui & W.H.Chen 1 | *O.* *hekouensis* (W.H.Chen & Y.M.Shui) Mich.Möller & A.Weber | China:Yunnan, Hekou county | Y.M. Shui et al. 94303-1 | KUN | This study | This study |
| *Ancylostemon hekouensis* Y.M.Shui & W.H.Chen 2 | *O.* *hekouensis* (W.H.Chen & Y.M.Shui) Mich.Möller & A.Weber | China:Yunnan, Hekou county | Y.M. Shui et al. 94303-2 | KUN | This study | This study |
| *Ancylostemon humilis* W.T.Wang | *O.* *humilis* (W.T. Wang) Mich.Möller & A.Weber | China: Sichuan | R.H. Liang SC-YB | PE | GU350665 | GU350633 |
| *Ancylostemon mairei* (H.Lév.) Craib | *O.* *wangwentsaii* Mich.Möller & A.Weber | China: Yunnan | R.H. Liang, Y.N.-Qj | PE | GU350689 | GU350658 |
| *Ancylostemon rhombifolius* K.Y.Pan | *O.* *rhombifolia* (K.Y.Pan) Mich.Möller & A.Weber | China: Sichuan | R.H. Liang LRH-07-01 | PE | GU350664 | GU350632 |
| *Ancylostemon ronganensis* K.Y.Pan | *O.* *ronganensis* (K.Y.Pan) Mich.Möller & A.Weber | China: Guangxi, Rong'an county | M. Möller et al. MMO 06-776 | E | HQ632927 | HQ633023 |
| *Ancylostemon saxatilis* (Hemsl.) Craib 1 | *O.* *saxatilis* (Hemsl.) Mich.Möller & A.Weber | China: Chongqing, Nanchuan county | Y.M. Shui et al. B2012-128 | KUN | This study | This study |
| *Ancylostemon saxatilis* (Hemsl.) Craib 2 | *O.* *saxatilis* (Hemsl.) Mich.Möller & A.Weber | China: Chongqing, Nanchuan county | Y.M. Shui et al. B2012-102 | KUN | This study | This study |
| *Bournea leiophylla* (W.T.Wang) W.T.Wang & K.Y.Pan | *O.* *leiophylla* W.T.Wang | China: Fujian | X.R. Zhou ZXR-05-01 | PE | GU350676 | GU350644 |
| *Bournea sinensis* Oliv. | *O.* *sinensis* (Oliv.) Mich.Möller & A.Weber | China: Guangdong, Boluo county | M. Möller et al. MMO 08-1329 | E | HQ632912 | HQ633008 |
| *Briggsia chienii* Chun | *O.*  *chienii* (Chun) Mich.Möller & A.Weber | China: Zhejiang, Lin'an county | Y.M. Shui et al. B2012-016 | KUN | This study | This study |
| *Briggsia longifolia* Craib 1 | *O. longifolia* (Craib) Mich.Möller & A.Weber | China: Yunnan, Jingdong county | M. Möller et al. MMO 08-1239 | E | HQ632934 | HQ633030 |
| *Briggsia longifolia* Craib 2 | *O. longifolia* (Craib) Mich.Möller & A.Weber | China: Yunnan, Baoshan county | Z.Y. Yu et al. s.n. | KUN | This study | This study |
| *Briggsia muscicola* Craib | *O. muscicola* (Diels) Mich.Möller & A.Weber | unknown origin | Kew (ID1995-2229) | K | FJ501548 | FJ501366 |
| *Briggsia rosthornii* (Diels) B.L.Burtt 1 | *O. rosthornii* (Diels) Mich.Möller & A.Weber | China: Chongqing, Nanchuan county | Y.M. Shui et al. B2012-129 | KUN | This study | This study |
| *Briggsia rosthornii* (Diels) B.L.Burtt 2 | *O. rosthornii* (Diels) Mich.Möller & A.Weber | China: Guizhou, Jiangkou county | M. Möller et al. MMO 03-305 | E | This study | This study |
| *Briggsia rosthornii* var. *wenshanensis* K.Y.Pan | *O. rosthornii* var. *wenshanensis* (K.Y.Pan) Mich.Möller & A.Weber | China: Yunnan, Wenshan county | Y.M. Shui et al. B2012-100 | KUN | This study | This study |
| *Briggsia speciosa* (Hemsl.) Craib | *-* | China: Hupei, Enshi county | P. Zhou 2010-037 | E | This study | This study |
| *Briggsia stewardii* Chun | *O.* *stewardii* (Chun) Mich.Möller & A.Weber | China: Guangxi, Sanjiang county | M. Möller et al. MMO 06-917 | E | HQ632926 | HQ633022 |
| *Dayaoshania cotinifolia* W.T.Wang | *O.* *cotinifolia* (W.T.Wang) Mich.Möller & A.Weber | China: Guangxi, Jinxiu county | Q.M. Chuan 01 | IBK | HQ632914 | HQ633010 |
| *Deinocheilos jiangxiense* W.T.Wang | *O.*  *jiangxiensis* (W.T.Wang) Mich.Möller & A.Weber | China: Fujian, Jiangle county | M. Möller et al. MMO 09-1451 | E | HQ632933 | HQ633029 |
| *Isometrum eximium* Chun ex W.T.Wang & K.Y.Pan | *O. eximium* (Chun ex W.T.Wang & K.Y.Pan) Mich.Möller & A.Weber | China:Yunnan, Lijiang county | Y.M. Shui et al. B2012-132 | KUN | This study | This study |
| *Isometrum farreri* Craib | *O.*  *farreri* (Craib) Mich.Möller & A.Weber | China: Shaanxi, Mian county | P. Zhou ZP 2010-020 | E | JF697585 | JF697573 |
| *Isometrum lancifolium* (Franch.) K.Y.Pan | *O.* *lancifolia* (Franch.) Mich.Möller & A.Weber | China: Sichuan, Mianning county | M. Möller et al. MMO 09-1624 | E | HQ632924 | HQ633020 |
| *Isometrum lungshengense* (W.T.Wang) W.T.Wang & K.Y.Pan | *O.* *lungshengensis* (W.T.Wang) Mich.Möller & A.Weber | China: Guangxi, Longsheng county | M. Möller et al. MMO 06-916 | E | HQ632917 | HQ633013 |
| *Isometrum nanchuanicum* K.Y.Pan & Z.Y.Liu | *O.* *nanchuanica* (K.Y.Pan & Z. Y.Liu) Mich.Möller & A.Weber | China: Chongqing, Nanchuan county | Y.M. Shui et al. B2012-115 | KUN | This study | This study |
| *Isometrum primuliflorum* (Batalin) B.L.Burtt | *O.* *primuliflora* (Batalin) Mich.Möller & A.Weber | China: Sichuan, Danba county | M. Möller et al. MMO 09-1605 | E | HQ632923 | HQ633019 |
| *Opithandra acaulis* (Merr.) B.L.Burtt | *O.* *acaulis* (Merr.) Mich.Möller & A.Weber | China: Guangdong, Zhaoqing county | M. Möller et al. MMO 08-1328 | E | HQ632916 | HQ633012 |
| *Opithandra burttii* W.T.Wang | *O.* *burttii* (W.T.Wang) Mich.Möller & A.Weber | China: Guangdong, Wengyuan county | F. Wen 2010-05 | IBK | JF697582 | JF697570 |
| *Opithandra dalzielii* (W.W.Sm.) B.L.Burtt | *O.* *dalzielii* (W.W.Sm.) Mich.Möller & A.Weber | China: introduced from Guangdong, Shantou county | F. Wen 2010-06 | IBK | JF697583 | JF697571 |
| *Opithandra dinghushanensis* W.T.Wang | *O.* *dinghushanensis* (W.T.Wang) Mich.Möller & A.Weber | China: Guangdong, Zhaoqing county | Q.B. Lin LQB06-01 | PE | GU350675 | GU350643 |
| *Opithandra primuloides* (Miq.) B.L.Burtt 1 | *O.* *primuloides* (Miq.) Benth. & Hook.f. ex Clarke | Japan: Shikoku | G. Kokubugata 11869 | TNS | This study | This study |
| *Opithandra primuloides* (Miq.) B.L.Burtt 2 | *O.* *primuloides* (Miq.) Benth. & Hook.f. ex Clarke | Japan: unknown locality | T. Tsuzuki [cult. RBGE 19842178A] | E | FJ501546 | FJ501364 |
| *Opithandra sinohenryi* (Chun) B.L.Burtt | *O.* *sinohenryi* (Chun) Mich.Möller & A.Weber | China: Guangxi, Fangcheng county | M. Möller et al. MMO 07-1150 | E | HQ632913 | HQ633009 |
| *Oreocharis argyreia* Chun ex K.Y.Pan | - | China: Guangxi, Wuming county | M. Möller et al. MMO 07-1131 | E | HQ632919 | HQ633015 |
| *Oreocharis argyreia* var. *angustifolia* K.Y.Pan | - | China: Guangxi | R.H. Liang GX-SS-01 | PE | GU350671 | GU350639 |
| *Oreocharis aurea* Dunn 1 | - | China: Yunnan, Jinping county | M. Möller et al. MMO 06-980 | E | HQ632920 | HQ633016 |
| *Oreocharis aurea* Dunn 2 | - | China: Yunnan, Yuanyang county | Y.M. Shui et al. 83608 | KUN | This study | This study |
| *Oreocharis auricula* (S.Moore) C.B.Clarke | - | China: Guizhou, Jiangkou county | M. Möller et al. MMO 03-304 | E | FJ501481 | FJ501323 |
| *Oreocharis benthamii* C.B.Clarke | - | China: Guangdong, Zhaoqing county | M. Möller et al. MMO 08-1317 | E | JF697584 | JF697572 |
| *Oreocharis cinnamomea* J.Anthony | - | China: Yunnan, Ninglang county | Y.M. Shui et al. N600 | KUN | This study | This study |
| *Oreocharis cordatula* (Craib) Pellegr. | - | China: Yunnan, Yongsheng county | Y.M. Shui et al. B2012-092 | KUN | This study | This study |
| *Oreocharis dasyantha* var. *ferruginosa* K.Y.Pan | - | China: Hainan, Delong | Y.G. Wei 07-700 | E | HQ632918 | HQ633014 |
| *Oreocharis dayaoshanioides* Yan Liu & W.B.Xu | - | China: Guangxi, Wuzhou county | F. Wen 0901 | IBK | JF687580 | JF697568 |
| *Oreocharis dentata* A.L.Weitzman & L.E.Skog | - | China: Sichuan, Mt. Emei | Y.M. Shui et al. B2012-040 | KUN | This study | This study |
| *Oreocharis elliptica* J.Anthony | - | China:Yunnan, Deqin county (Meili) | Y.M. Shui et al. D255 | KUN | This study | This study |
| *Oreocharis georgei* J.Anthony | - | China: Yunnan, Ninglang county | Y.M. Shui et al. N569 | KUN | This study | This study |
| *Oreocharis henryana* Oliv. 1 | - | China: Sichuan, Lushan county | M. Möller et al. MMO 10-1691 | E | JF697586 | JF697574 |
| *Oreocharis henryana* Oliv. 2 | - | China: Sichuan, Mt. Emei | Y.M. Shui et al. B2012-034 | KUN | This study | This study |
| *Oreocharis hirsuta* Barnett | - | Thailand, Chiang Mai | D. Middleton 4550 | E | This study | This study |
| *Oreocharis jinpingensis* W.H.Chen & Y.M.Shui | - | China:Yunnan, Jinping county | Y.M. Shui et al. 90966 | KUN | This study | This study |
| *Oreocharis magnidens* Chun ex K.Y.Pan | - | China: Guangxi, Jinxiu county | M. Möller et al. MMO 06-896 | E | HQ632930 | HQ633026 |
| *Oreocharis rotundifolia* K.Y.Pan | - | China: Yunnan, Pingbian county | Y.M. Shui et al. 92334 | KUN | This study | This study |
| *Oreocharis xiangguinensis* W.T.Wang & K.Y.Pan | - | China: Guangxi, Lingui county | M. Möller et al. MMO 05-741 | E | HQ632932 | HQ633028 |
| ***Paraisometrum mileense* W.T.Wang Y1** | *O.* *mileensis* (W.T.Wang) Mich.Möller & A.Weber | China: Yunnan, Shilin county | Y.M. Shui et al. 64957-1 | KUN | This study | This study |
| ***Paraisometrum mileense* W.T.Wang Y2** | *O.* *mileensis* (W.T.Wang) Mich.Möller & A.Weber | China: Yunnan, Shilin county | Y.M. Shui et al. 64957-2 | KUN | This study | This study |
| ***Paraisometrum mileense* W.T.Wang Gx11a** | *O.* *mileensis* (W.T.Wang) Mich.Möller & A.Weber | China: Guangxi, Longlin county | Y.M. Shui et al. B2010-025-1 | KUN | This study | This study |
| ***Paraisometrum mileense* W.T.Wang Gx11b** | *O.* *mileensis* (W.T.Wang) Mich.Möller & A.Weber | China: Guangxi, Longlin county | Y.M. Shui et al. B2010-025-2 | KUN | This study | This study |
| ***Paraisometrum mileense* W.T.Wang Gx11h** | *O.* *mileensis* (W.T.Wang) Mich.Möller & A.Weber | China: Guangxi, Longlin county | Y.M. Shui et al. B2010-025-3 | KUN | This study | This study |
| ***Paraisometrum mileense* W.T.Wang Gz12a** | *O.* *mileensis* (W.T.Wang) Mich.Möller & A.Weber | China: Guizhou, Xingyi county | Y.M. Shui et al. B2010-036-1 | KUN | This study | This study |
| ***Paraisometrum mileense* W.T.Wang Gz12c** | *O.* *mileensis* (W.T.Wang) Mich.Möller & A.Weber | China: Guizhou, Xingyi county | Y.M. Shui et al. B2010-036-2 | KUN | This study | This study |
| ***Paraisometrum mileense* W.T.Wang Gz12i** | *O.* *mileensis* (W.T.Wang) Mich.Möller & A.Weber | China: Guizhou, Xingyi county | Y.M. Shui et al. B2010-036-3 | KUN | This study | This study |
| *Thamnocharis esquirolii* (H.Lév.) W.T.Wang | *O.* *esquirolii* H.Lév. | China: Guizhou, Anlong county | D.W. Zhang 723 | IBK | HQ632915 | HQ633011 |
| *Tremacron aurantiacum* K.Y.Pan | *O.* *pankaiyuae* Mich.Möller & A.Weber | China: unknown locality | [cult. RBGE 20060865] | E | HQ632925 | HQ633021 |
| *Tremacron begoniifolium* H.W.Li 1 | *O.* *begoniifolia* (H.W.Li) Mich.Möller & A.Weber | China:Yunnan, Jingdong county | M. Möller et al. MMO 08-1221 | E | HQ632929 | HQ633025 |
| *Tremacron begoniifolium* H.W.Li 2 | *O.* *begoniifolia* (H.W.Li) Mich.Möller & A.Weber | China: Yunnan, Yuanyang county | Y.M. Shui et al. 85538 | KUN | This study | This study |
| *Tremacron forrestii* Craib | *O.* *craibii* Mich.Möller & A.Weber | China: Sichuan, Panzhihua city | M. Möller et al. MMO 07-1072 | E | HQ632921 | HQ633017 |
| *Tremacron urceolatum* K.Y.Pan 1 | *O.* *urceolata* (K.Y.Pan) Mich.Möller & A.Weber | China: Sichuan, Liangshan county | M. Möller et al. MMO 09-1633 | E | HQ632922 | HQ633018 |
| *Tremacron urceolatum* K.Y.Pan 2 | *O.* *urceolata* (K.Y.Pan) Mich.Möller & A.Weber | China: Yunnan, Ninglang county | Y.M. Shui et al. N606 | KUN | This study | This study |
