# Supplementary material for: Taxonomic Status, Phylogenetic Affinities and Genetic Diversity of a Presumed Extinct Genus, Paraisometrum W.T. Wang (Gesneriaceae) from the Karst Regions of Southwest China
Source: PLoS One. 2014 Sep 24;9(9):e107967. doi: 10.1371/journal.pone.0107967 (PMC4176718; doi:10.1371/journal.pone.0107967)
Supplement: Table S2 — Characteristics of the Bayesian inference analysis of 86 samples of combined ITS and trn LF data. (DOC) [file pone.0107967.s007.doc]

**Supporting Information**

**Table S2: Characteristics of the Bayesian inference analysis of 86 samples of combined ITS and *trn*LF data.**

Average standard deviation of split frequencies: 0.009025

Acceptance rates for the moves in the "cold" chain of run 1:

With prob. (last 100) chain accepted proposals by move

35.5 % ( 18 %) Dirichlet(Revmat{1})

45.2 % ( 31 %) Slider(Revmat{1})

24.9 % ( 18 %) Dirichlet(Revmat{2})

30.7 % ( 31 %) Slider(Revmat{2})

29.2 % ( 26 %) Dirichlet(Revmat{3})

58.7 % ( 50 %) Slider(Revmat{3})

25.9 % ( 27 %) Dirichlet(Pi{1})

26.1 % ( 27 %) Slider(Pi{1})

23.8 % ( 28 %) Dirichlet(Pi{2})

25.7 % ( 29 %) Slider(Pi{2})

24.5 % ( 25 %) Multiplier(Alpha{1})

26.1 % ( 23 %) Multiplier(Alpha{2})

26.5 % ( 25 %) Multiplier(Alpha{3})

20.0 % ( 14 %) ExtSPR(Tau{all},V{all})

7.6 % ( 7 %) ExtTBR(Tau{all},V{all})

26.7 % ( 22 %) NNI(Tau{all},V{all})

13.6 % ( 23 %) ParsSPR(Tau{all},V{all})

26.2 % ( 21 %) Multiplier(V{all})

38.1 % ( 34 %) Nodeslider(V{all})

24.6 % ( 33 %) TLMultiplier(V{all})

Acceptance rates for the moves in the "cold" chain of run 2:

With prob. (last 100) chain accepted proposals by move

35.3 % ( 26 %) Dirichlet(Revmat{1})

45.3 % ( 31 %) Slider(Revmat{1})

24.8 % ( 28 %) Dirichlet(Revmat{2})

30.8 % ( 18 %) Slider(Revmat{2})

29.7 % ( 23 %) Dirichlet(Revmat{3})

58.2 % ( 41 %) Slider(Revmat{3})

25.9 % ( 26 %) Dirichlet(Pi{1})

26.6 % ( 22 %) Slider(Pi{1})

24.1 % ( 29 %) Dirichlet(Pi{2})

25.8 % ( 27 %) Slider(Pi{2})

24.1 % ( 25 %) Multiplier(Alpha{1})

26.1 % ( 29 %) Multiplier(Alpha{2})

26.1 % ( 28 %) Multiplier(Alpha{3})

20.1 % ( 19 %) ExtSPR(Tau{all},V{all})

7.6 % ( 9 %) ExtTBR(Tau{all},V{all})

26.8 % ( 25 %) NNI(Tau{all},V{all})

13.7 % ( 10 %) ParsSPR(Tau{all},V{all})

26.2 % ( 26 %) Multiplier(V{all})

38.4 % ( 39 %) Nodeslider(V{all})

24.5 % ( 29 %) TLMultiplier(V{all})

Chain swap information for run 1:

1 2 3 4

----------------------------------

1 | 0.25 0.02 0.00

2 | 333481 0.19 0.00

3 | 333653 333161 0.16

4 | 332620 333736 333349

Chain swap information for run 2:

1 2 3 4

----------------------------------

1 | 0.25 0.02 0.00

2 | 333152 0.19 0.01

3 | 333430 333387 0.16

4 | 333206 333371 333454

Upper diagonal: Proportion of successful state exchanges between chains

Lower diagonal: Number of attempted state exchanges between chains

Chain information:

ID -- Heat

-----------

1 -- 1.00 (cold chain)

2 -- 0.91

3 -- 0.83

4 -- 0.77

Heat = 1 / (1 + T * (ID - 1))

(where T = 0.10 is the temperature and ID is the chain number)
